# Interplay between splicing and transcriptional pausing exerts genome-wide control over alternative polyadenylation

**DOI:** 10.1080/21541264.2021.1959244

**Published:** 2021-08-07

**Authors:** Carmen Mora Gallardo, Ainhoa Sánchez de Diego, Carlos Martínez-A, Karel H.M. van Wely

**Affiliations:** Department of Immunology and Oncology Centro Nacional De Biotecnología (CNB)/, CSIC Darwin 3, Campus UAM Cantoblanco, Madrid, Spain

**Keywords:** RNA splicing, polyadenylation, transcription pausing, sequence analysis, mammalian gene expression

## Abstract

Recent studies have identified multiple polyadenylation sites in nearly all mammalian genes. Although these are interpreted as evidence for alternative polyadenylation, our knowledge of the underlying mechanisms is still limited. Most studies only consider the immediate surroundings of gene ends, even though *in vitro* experiments have uncovered the involvement of external factors such as splicing. Whereas *in vivo* splicing manipulation was impracticable until recently, we now used mutants in the *Death Inducer Obliterator* (*DIDO*) gene to study their impact on 3ʹ end processing. We observe multiple rounds of readthrough and gene fusions, suggesting that no arbitration between polyadenylation sites occurs. Instead, a window of opportunity seems to control end processing. Through the identification of T-rich sequence motifs, our data indicate that splicing and transcriptional pausing interact to regulate alternative polyadenylation. We propose that 3ʹ splice site activation comprises a variable timer, which determines how long transcription proceeds before polyadenylation signals are recognized. Thus, the role of core polyadenylation signals could be more passive than commonly believed. Our results provide new insights into the mechanisms of alternative polyadenylation and expand the catalog of related aberrations.

**Abbreviations** APA: alternative polyadenylation; bp: basepair; MEF: mouse embryonic fibroblasts; PA: polyadenylation; PAS: polyadenylation site; Pol II: (RNA) polymerase II ; RT-PCR:reverse-transcriptase PCR; SF:splicing factor; SFPQ:splicing factor rich in proline and glutamine; SS:splice site; TRSM:Thymidine rich sequence motif; UTR:untranslated terminal region

## Introduction

Formation of the 3ʹ tail of messenger RNA by cleavage and polyadenylation (PA) is an essential step that aides export from the nucleus and translation in the cytoplasm. PA requires recognition of the 3ʹ gene end by a multiprotein complex that catalyzes cleavage of the precursor, its release from RNA Polymerase II (Pol II), and poly(A) tail addition by a dedicated polymerase [1]. Finally, an exonuclease promotes release of the remaining transcription complex from the chromatin template [2]. Most of the genes transcribed by RNA Pol II lack a defined 3ʹ border and produce multiple ends [3]. This phenomenon, termed alternative polyadenylation (APA) is an important regulator of mRNA isoform expression [4]. APA has been associated with important biological processes such as development, cell differentiation, and proliferation [5]. Altered APA patterns also characterize several pathological states, for example shortening of 3ʹ untranslated regions (UTRs) during oncogenic dedifferentiation [6,7]. Much effort has recently gone into cataloging polyadenylation sites (PAS), which has given rise to a comprehensive census and has identified APA in more than half of all mammalian genes [8]. While the mechanisms of RNA cleavage and poly(A) tail addition are currently being elucidated, many details of polyadenylation site (PAS) selection and APA remain poorly understood.

The majority of recent studies on polyadenylation focused on cis-acting elements [3,9]. For example, gradual decrease of 3ʹ end processing factors during early embryonic development produces slippage toward downstream PAS [6]. More distant genomic features are considered important, too, since PA is coordinated with processing of the upstream RNA splice signals [10,11]. *In vitro* experiments indicate that recognition of the upstream 3ʹ splice site (SS) is of particular importance, and that binding of the splicing apparatus defines the constraints of PA [12,13]. Analyses *in vivo* have shown that terminal 3ʹ SS processing is sufficiently rapid for selection of canonical PAS for most genes [11]. PA is assisted further by Pol II pausing at the 3ʹ gene end, again observed for a large subset of genes [14]. This Pol II pausing frequently occurs some distance downstream of the actual gene body, so a stretch of RNA is exposed to promote assembly of the actual 3ʹ end processing factors [15,16]. Finally, Pol II pausing is thought to have an integrating role, allowing distant regions on the precursor RNA to be processed together [17]. Notwithstanding the widespread observations of Pol II pausing and processing factor recruitment around PAS, the flexibility of APA is understood incompletely. We show here that Pol II pausing occurs frequently, but may not be a universal feature of 3ʹ gene ends. Transcriptional pausing is enforced by T-rich sequence motifs (TRSM) in the template DNA *in vitro* [18]. However, a genome-wide analysis of TRSM at 3ʹ gene ends has not yet been carried out. Our prior work has identified an interplay between TRSM and mutants of the *Death Inducer Obliterator* (*DIDO*) gene. *DIDO* has a role in recruiting splicing factors (SF) to 3ʹ SS, and deletion of different sections of *DIDO* lead to splicing defects that are differentially modulated by TRSM [19].

*DIDO* is the vertebrate homolog to yeast BYE1 and insect PPS genes [20,21], and produces a family of transcription factors that bind to histone H3 trimethylated on lysine 4 [22,23]. The protein family also binds to the jaw and funnel domains of RNA Pol II [21], leading to a bivalent genomic distribution at both gene ends [24,25]. The vertebrate *DIDO* gene produces several protein products (DIDO1, −2, and −3) by alternative splicing. Among these, only DIDO3 catalyzes recruitment of the splicing factor SFPQ to RNA precursors [19]. Since SFPQ binds upstream of 3ʹ splice sites (3ʹ SS), where it controls U2 spliceosome positioning [26], DIDO3 forms a bridge between RNA Pol II and the splicing apparatus. Elimination of a DIDO3-specific exon (E16) allows for production of DIDO2, an isoform that binds Pol II but fails to enrich SFPQ [19,24]. While the E16 mutant suffers from an overall decrease of splicing capacity, a group of SS that are flanked by TRSM are processed with increased efficiency. A second *DIDO* mutant (E3+4), which lacks histone binding but retains the domains for RNA Pol II and SFPQ interaction, also has been generated [27]. Compared to E16, E3+4 shows mild splicing defects but is more responsive to sequence bias. E3+4 is particularly sensitive to TRSM, which in this mutant greatly increase RNA processing [19].

Here, we combine our prior knowledge of *DIDO* mutants with a statistical analysis of APA. We identified distant TRSM that stimulate PAS usage, and compared these to our data on 3ʹ SS usage [19]. Where prior publications reported only PAS usage frequencies [28,29], the RNA sequencing of our mutants allowed us to establish a statistical correlation between sequence motifs, Pol II pausing, and PAS readthrough. Instead of arbitration between adjacent PAS, a window of opportunity appears to determine the use or readthrough of individual signals. Terminal 3ʹ SS and sequence motifs have an important role in this process, indicating that TRSM comprise a timer to resolve terminal exon splicing and thereby control PAS selection. Differential positioning of critical pause sites suggests that multiple routes may establish coupling between splicing and polyadenylation.

## Results

### T-rich sequences are abundant but heterogeneously distributed

**Figure 1.**
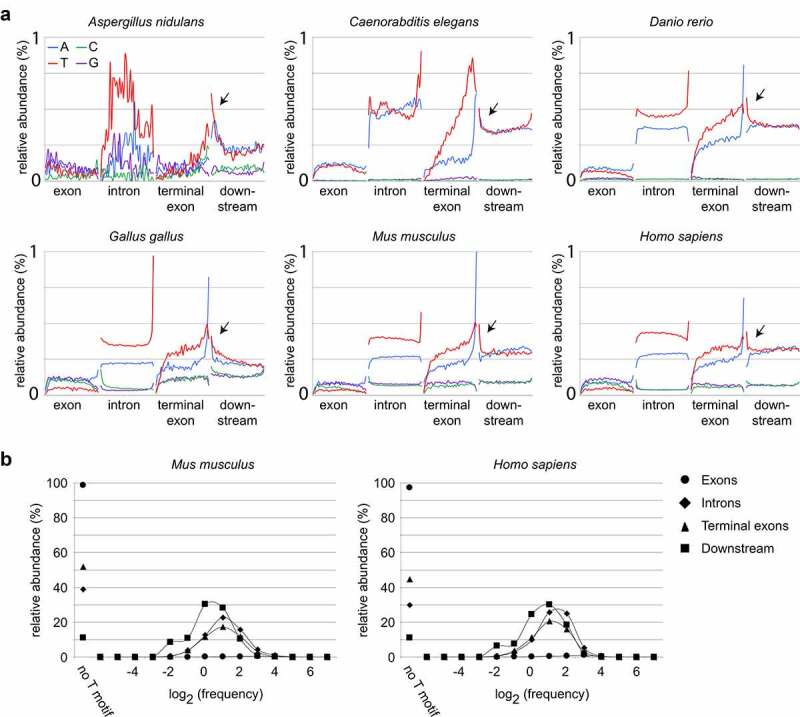
**TRSM occur frequently in metazoan genomes**. A sliding window was used to scan for short sequences bearing more than 75% of a single base. (a) Relative abundance of short repetitive sequences according to genomic feature shows enrichment of A-rich and T-rich sequences in non-coding regions. In all cases, TRSM are more abundant than repeats of other bases. Note the increase of TRSM at the end of introns (polypyrimidine tract) and around polyadenylation sites (arrows). (b) TRSM are unevenly distributed. Regions downstream of a small but detectable proportion of genes are devoid of TRSM, and TRSM frequency in other genes may vary by several orders of magnitude

Our recent data uncovered a role for DIDO3 in transcription, and indicated that the interplay between DIDO3 levels and intronic Thymidine-rich sequence motifs controls exon skipping or inclusion [19]. In model transcripts, T-rich sequences with a length of 5 to 10 basepairs (bp) are efficient and selective inducers of RNA pol II pausing; such pause sites are thought to promote integration of multiple regulatory signals [18,30]. To determine if strongly biased sequences could have a role across a large subset of genes, we scanned the genomes of several vertebrates and non-vertebrate organisms (Figure 1(a)). Briefly, a sliding window was used to identify 12 base-pair (bp) regions bearing at least 75% Thymidine in all known internal exons, introns, terminal exons, as well as directly downstream of described genes. As control, the same scan was carried out for the other bases (Adenosine, Cytidine, and Guanosine).

Scanning of genomic regions showed that short repetitive regions are exceptionally abundant in introns, and in metazoans mostly correspond to A- and T-rich sequences. T-rich sequence motifs (TRSM) are most abundant toward the 3ʹ end of introns, since they make up a large proportion of polypyrimidine tracts. In addition to introns, terminal exons and extragenic regions downstream of genes also showed abundant A- and T-rich sequences. A-rich sequences were found frequently at the 3ʹ end of terminal exons, where they correspond to polyadenylation signals. Remarkably, the abundance of T-rich sequences, too, increased toward the 3ʹ end of terminal exons, but in addition showed a high frequency at the 5ʹ region of intergenic regions (Figure 1(a), arrows). Except for the extreme 3ʹ end of terminal exons, T-rich sequences occurred more frequently than repeats in other bases throughout the terminal exon and immediately downstream of genes.

Next, we determined the proportion of each gene feature containing T-rich sequences in mammals (Figure 1(b)). Scoring for the presence of one or more copies of the T-rich sequence showed an uneven distribution; while the vast majority of internal exons was devoid of TRSM, only a small proportion of non-coding features bore no TRSM at all. A considerable proportion of non-coding genetic features thus included one or more potential Pol II pause sites, albeit with different frequencies. Remarkably, a high proportion of intergenic regions immediately downstream of genes contained TRSM, with a small proportion without any of these. TRSM thus occur frequently in the genomes of a variety of organisms but are unevenly distributed among genes. The high proportion of downstream regions bearing TRSM suggests a regulatory role in 3ʹ end processing.

### Contribution of T-rich sequences to Pol II pausing

**Figure 2.**
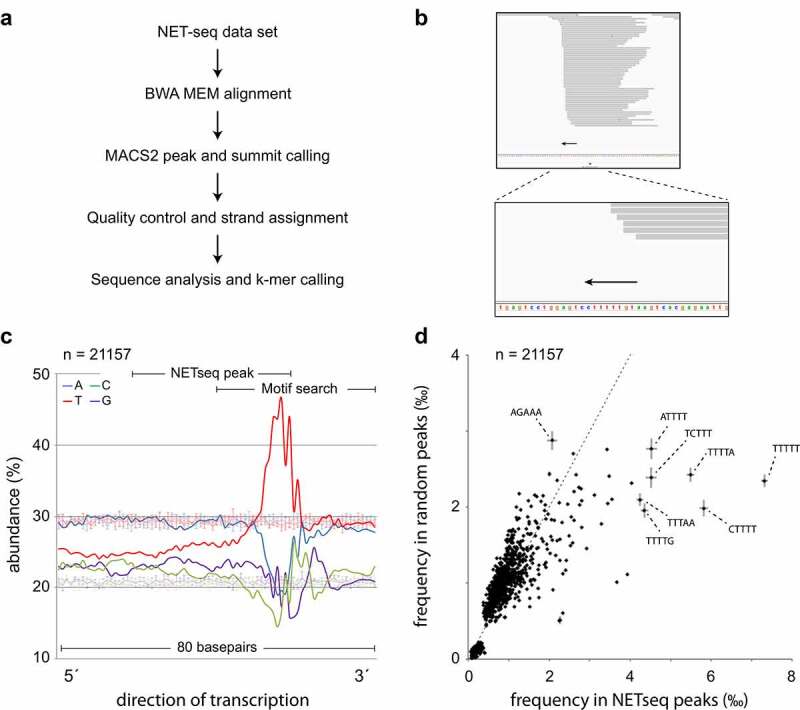
**TRSM contribute to RNA Pol II pausing**. (a) Depiction of the analysis pipeline. Details are given in the Methods section. (b) Example of a TRSM in a MACS2 peak. Arrows indicate TRSM in agreement with direction of transcription. See supplementary figure S1 for additional examples. (c) Thymidine enrichment (red line) on the 3´ flank of NET-seq peaks. A 5 × 5000 sample of random regions (thin lines) was used as control and for calculation of standard deviation (error bars). Bases are colored as in Figure 1. (d) Identification of 5-mer sequences enriched on the 3´ flank. Enriched 5-mers appear to the right of the diagonal line; the further to the right from the diagonal, the higher the enrichment. Error bars (subset) are derived from a 5 × 5000 random sample

Since T-rich repeats in the template produce a paused state in RNA polymerases *in vitro* [18], they are thought to promote splicing by providing additional time for the recruitment of RNA processing factors. For example, we have shown that sequence bias modulates DIDO3-dependent recruitment of SFPQ to 3ʹ SS [19]. To evaluate the contribution of sequence motifs to Pol II pausing at the 3ʹ end of genes *in vivo*, we analyzed NET-seq data [31] which allowed for high-resolution directional Pol II mapping [32]. Briefly, reads were aligned to the *Mus musculus* genome and peaks assigned. Since exon-bound nucleosomes may cause Pol II stalling [33], peaks overlapping with internal but not terminal exons were filtered out. Finally, sequence composition of the entire peak and frequency of all possible 5-mer sequence motifs on the downstream flank were analyzed (Figure 2(a)).

Sequence composition analysis of the peaks and immediate surroundings revealed a marked increase of Thymidine frequency on the 3´ flank (Figure 2(c)), typically overlapping with a sharp cut in the newly synthetized RNA (Figure 2(b)). To further analyze 3´ flanks, the frequency of short sequence motifs in this regions was determined. Among all possible combinations of 5-mers, a small subset containing multiple Thymidines was highly enriched (Figure 2(d)). Thus, short Thymidine-rich sequences were commonly associated with Pol II pause sites. While these sequences temporarily paused Pol II, they probably did not fully stop transcription, as closely spaced tandem peaks were also found (supplementary figure S1).

In highly transcribed genes such as ACTB and EIF1, sharply defined spikes typically generated by NET-seq seem to fuse into broader peaks (supplementary figure S2). Continued gene expression might thus cause stalling of transcriptional complexes further upstream, comparable to cars in a traffic jam. As DIDO3 associates with RNA Pol II during transcription [21,24], this protein also distributed upstream of TRSM (supplementary figures S2-S4). In general, DIDO3 peaks were broader because of resolution limitations of CHIP-seq. Still, the downstream flank of these peaks was defined by TRSM. Strikingly, both NET-seq and CHIP-seq signals were found a considerable distance downstream of genes. Accordingly, a considerable proportion of known PAS sites [8] located to the so-called “junk DNA”.

### Altered PAS usage in DIDO mutants

**Figure 3.**
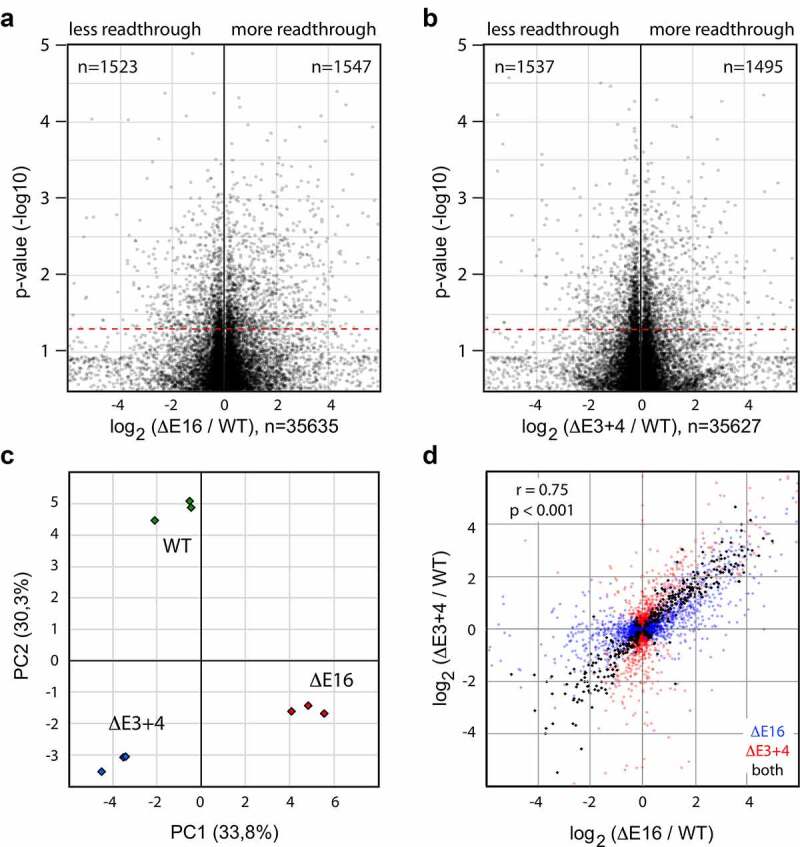
**Altered PAS usage in *DIDO* mutants**. (a,b) Volcano plots showing overall effect of *DIDO* mutation on PAS usage. Readthrough levels were determined for all expressed PAS, and compared between *DIDO* mutants and wild-type controls. E16 (a) and E3+4 (b) are shown separately. In both mutants, approximately 10% of PAS show significantly (*p* < 0.05, red line) altered readthrough levels. (c) Principle component analysis separated WT, E16 and E3+4 into discrete groups. While both mutants showed a similar displacement along PC2, their PC1 shift was opposite. (d) Direct comparison of readthrough in E16 and E3+4. The majority of PAS that show significantly altered readthrough as compared to WT controls show similar behavior in the two mutants

While analysis of individual model transcripts indicates a role for the terminal intron in polyadenylation, the global impact of splicing on APA remains largely unknown. To address this question, we used *DIDO* gene mutants, which suffer from splicing defects due to altered 3ʹ SS usage [19]. To evaluate readthrough, *DIDO* RNA sequencing data were combined with PolyASite, a comprehensive database of experimentally identified PAS [8]. Readthrough was defined as the number of downstream reads divided by the number of upstream reads, each obtained from a 275 bp window. For every PAS, transcriptional readthrough was calculated in triplicate, and the *DIDO* mutant value compared to wild-type (WT) controls. After filtering out low expression genes, PAS showing significantly altered readthrough were reported. Volcano plots of E16 (Figure 3(a)) and E3+4 (Figure 3(b)) mutants, representing readthrough levels relative to WT controls, show significantly altered usage of several thousand PAS.

Clustering of readthrough by principal component analysis (PCA) produced three distinct sample groups corresponding to WT, E16 and E3+4 (Figure 3(c)), in a distribution that closely resembles the PCA for exon skipping [19]. Compared to WT controls, both E3+4 and E16 were displaced along one axis (PC2). The shift along the other axis (PC1) was most notable for E16, indicating that the two mutants share some alterations but also produce unique polyadenylation events. As PCA accentuates differences between sample groups but underestimates possible similarities, we calculated readthrough levels for all PAS showing significantly altered behavior in both mutants. Next, readthrough levels relative to WT controls were determined and the data of each mutant plotted on separate axes. The majority (90%) of PAS significantly altered in both mutants showed shifted in the same direction in E16 and E3+4 (Figure 3(d), black dots). Next, we calculated readthrough for PAS that showed significantly altered behavior in one mutant only, and plotted the data for each mutant on separate axes (Figure 3(d), colored dots). Readthrough levels relative to WT controls showed substantial correlation between E3+4 and E16; nearly 70% of PAS behaved similarly in both mutants when changes were significant for one condition only. The behavior of approximately 30% of PAS was opposite, showing an increase in one *DIDO* mutant but decrease in the other.

While the contribution of the upstream 3ʹ SS to end processing has been evaluated *in vitro* for a few substrates, possible global effects of altered splicing on a broad range of RNA precursors remain to be determined. We thus looked for parameters that distinguish the PAS showing altered levels of readthrough in the *DIDO* mutants from the general population. To evaluate overall behavior of PAS, a classification according to readthrough and expression levels was performed (supplementary figure S5). The majority of PAS (128.023 out of 188.695, 68%) showed no readthrough under any condition or corresponded to low expression genes in MEF, and thus were filtered out. PAS that were affected by *DIDO* mutation followed a pattern comparable to the general distribution; the majority of these showed approximately 50% readthrough in WT MEF (supplementary figure S5A). *DIDO* mutation thus affects PAS already susceptible to readthrough under WT conditions, as observed before for exon skipping [19]. Less than 1% of PAS showing altered readthrough were found downstream of single-exon genes without upstream splice site. Expression levels of the genes located directly upstream of affected PAS followed a distribution comparable to the general population (supplementary figure S5B). Thus, altered gene expression unlikely accounted for the observed readthrough in *DIDO* mutants.

### Altered PAS usage correlates with sequence composition

**Figure 4.**
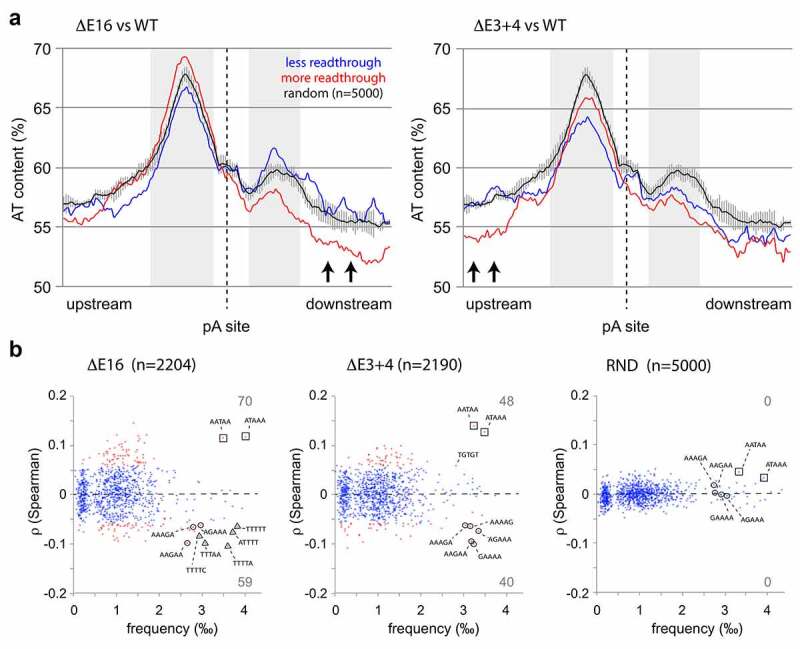
**Relation between sequence composition and PAS readthrough**. (a) Analysis of AT content of genomic regions immediately surrounding PAS. Overall AT content was analyzed for all PAS undergoing significantly increased (red) or decreased (blue) readthrough in E16 (left) and E3+4 (right). A region of 75 bases upstream and downstream of each site is depicted. (b) Identification of 5-mer motifs in the genomic regions surrounding PAS. Horizontal axis depict the overall frequency of occurrence of each 5-mer, and vertical axis Spearman correlation with readthrough. Motifs showing significant correlation are depicted in red. Controls were derived from a 5 × 1000 random sample

The nucleotide composition of PAS has been the focus of several studies, and has led to the identification of sequence motifs located immediately upstream and downstream of the canonical poly(A) signal [8,34,35]. Independently, the search for 3ʹ end processing factors has identified protein interaction that recognize these cis-acting signals when they emerge from the transcription complex [3]. To identify core sequence motifs that modulate polyadenylation, a 150 basepair window surrounding alternatively used PAS was analyzed (Figure 4). PAS were grouped according to significantly decreased or increased readthrough in the *DIDO* mutants, and compared to random controls. As Adenosine and Thymidine bases show a high abundance in this region (Figure 1), we first focused on overall AT (or AU) content (Figure 4(a)). Overall AT content of the core poly(A) signal containing the canonical AATAAA was similar for all conditions. The composition in flanking regions that harbor cis-acting sequence elements however differed between the three sample groups. For the upstream cis-acting element, high AT content favors readthrough while for the downstream region it favors processing. In addition to the cis-acting elements, AT content analysis showed differences in the regions located further up- and downstream (Figure 4(a), arrows). Here, the two mutants showed divergent behavior; while E16 was particularly sensitive for sequence bias downstream of the PAS, E3+4 showed particular selectiveness for bias located at least 150 bases upstream.

Because a series of interrelated sequence motifs determines recognition by cis-acting polyadenylation factors [3], we screened the differentially used PAS for all combinations of short 5-mer sequences. First, overall occurrence of all possible 5-mers in the set of differentially used PAS was calculated. Next, the frequency of every 5-mer in individual PAS was combined with the fold change in readthrough of the same PAS. This allowed us to calculate – by Spearman correlation – the statistical significance of the contribution of each 5-mer to PAS usage (Figure 4(b)). A random selection of PAS without altered behavior in *DIDO* mutants served as control.

In agreement with prior [28,29] reports, 5-mers comprising the canonical AATAAA poly(A) signal were amongst the most common motifs detected (Figure 4(b), squares). Strikingly, presence of these motifs, as well as 5-mers mapping to GU-rich core signals [3], correlated with increased readthrough in the *DIDO* mutants. While these motifs appeared with a comparable frequency in a random control group, here they did not bear significant correlation to readthrough.

Matching the overall AT content of the wider PAS architecture, a set of T-rich motifs was included in the 5-mers in E16 that correlated negatively with readthrough in E16 (Figure 4(b), triangles). While these motifs might correspond to cis-acting sequences [3], their considerable distance (200 bases) from the central PAS indicates that they belong to a separate group of signals. Finally, several motifs without enrichment in NET-seq (AGAAA, Figure 2(d)) showed significant negative correlation with readthrough (Figure 4(b), circles). These latter motifs combined into the alternative PA signal AAGAAA [28,29], indicating a shift from canonical to alternative PAS in the *DIDO* splicing mutants. Together, the effect of flanking sequence motifs and use of alternative sites suggest that APA in *DIDO* mutants is modulated by signals outside the PAS core.

### Common TRSM correlate with splicing and polyadenylation

**Figure 5.**
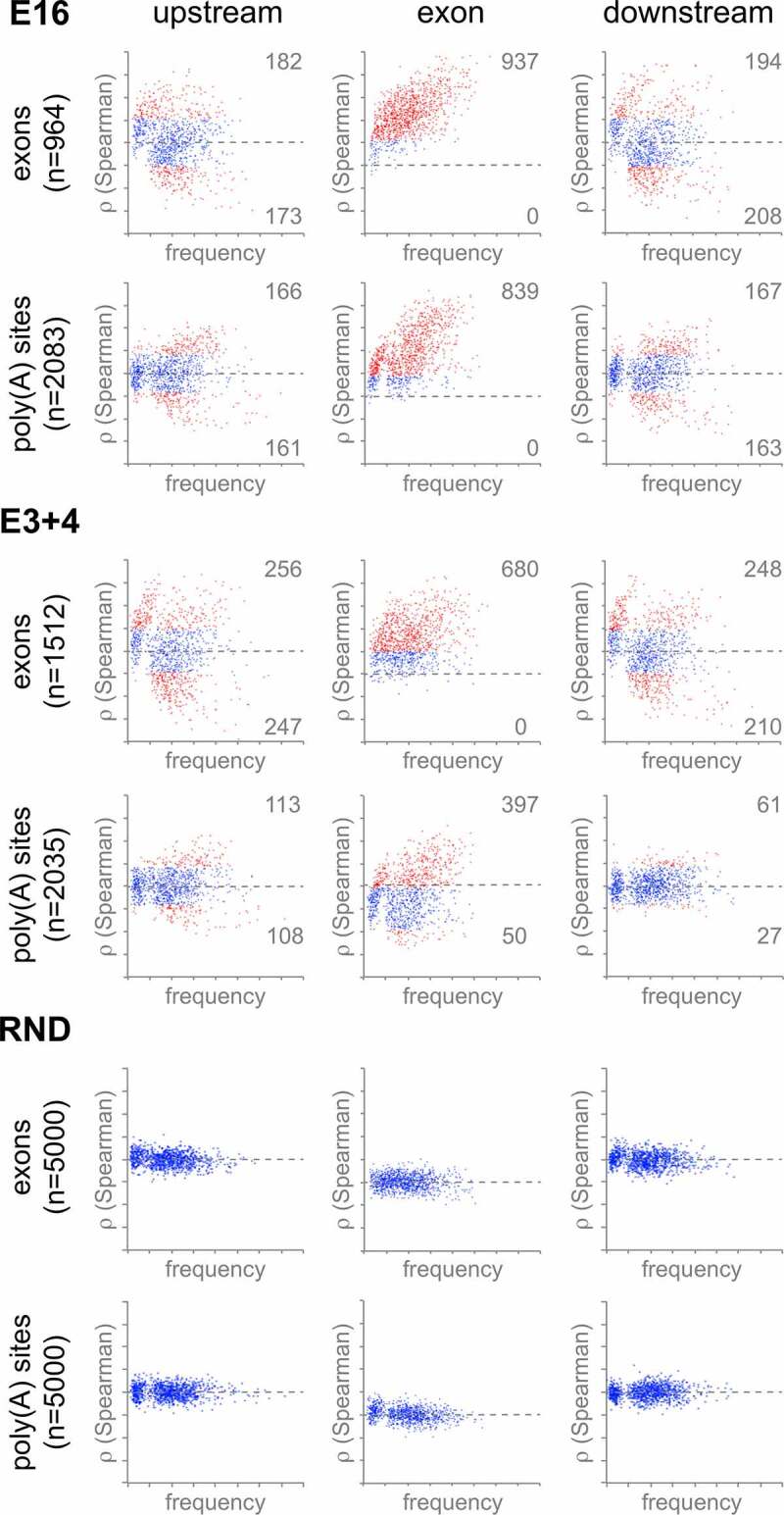
**Identification of readthrough-associated sequence motifs**. Scatter plots showing frequency of 5-mer sequences surrounding the terminal exon (upper rows) or PAS (lower rows). Horizontal axes depict the overall frequency of occurrence, and vertical axes Spearman correlation with readthrough. Horizontal dashed lines indicate no change in exon skipping or readthrough. Motifs showing significant correlation with readthrough are depicted in red. Controls were derived from a 5 × 1000 random sample. Individual 5-mer motifs are annotated in supplementary figures S6-S8

To test the hypothesis that a wider gene architecture controls polyadenylation, regions spanning the entire terminal exon were analyzed in the same way as the PAS core. Because of the scattered distribution of NET-seq summits, regions up- and downstream were also included (Figure 5). Frequencies of 5-mers were determined separately for upstream regions, exons scaled to 200 bp, and downstream regions. Statistical significance of the effect of each motif on was again calculated by Spearman correlation; we linked the frequency of each 5-mer in an exon to fold change in skipping. Finally, the same correlation coefficients were calculated to measure the effect of 5-mers on PAS readthrough. In this case, terminal exons were defined as the region between the closest upstream 3ʹ SS and the PAS. Random selections of internal exons and PAS not significantly affected in *DIDO* mutants served as negative controls.

Skipping of internal exons in the *DIDO* mutants showed negative correlation with 15–20% of upstream and downstream 5-mers, in particular with T-rich motifs (supplementary figures S6–S7). Again, a random control population showed no significant correlation (supplementary figure S8). As sequence composition in coding regions is dominated by amino-acid composition, few TRSM favor inclusion of internal exons. UTR, not limited by amino-acid composition, show greater variability of sequence motifs. Even so, the E16 mutant does not seem to be able to make use of UTR-based sequence motifs, as terminal exons showed a motif correlation pattern that is comparable to internal exons (supplementary figure S6). In contrast to E16, the partially functional E3+4 showed significant negative correlation between readthrough and a subset of AT-rich motifs in terminal exons (supplementary figure S7B). Although regions downstream of PAS produced a low number of significant correlating motifs, overall Spearman correlation in E3+4 was much lower than observed in the corresponding region of internal exons.

**Figure 6.**
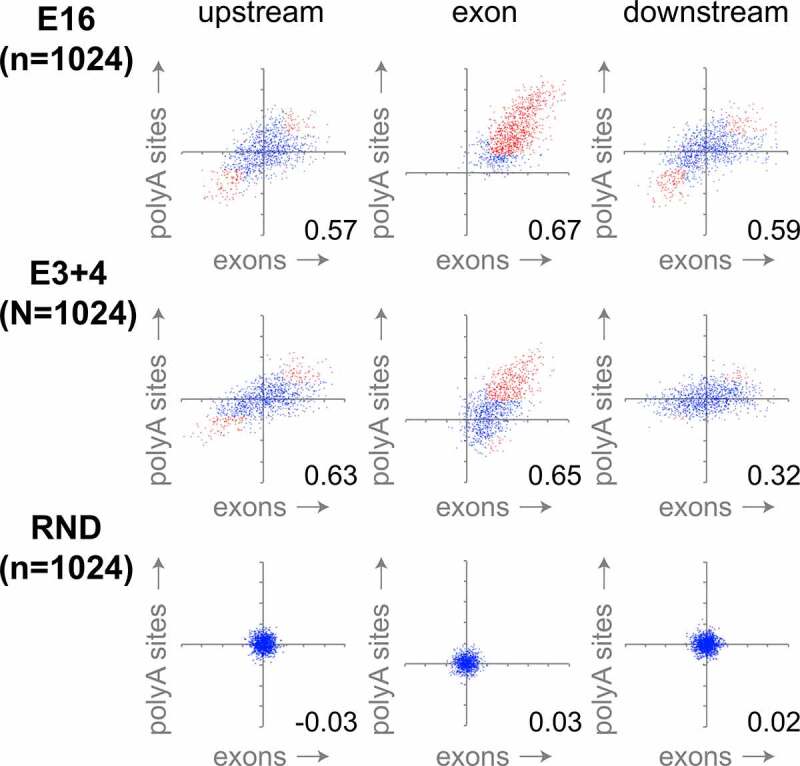
**Exon skipping and PAS readthrough are affected by a common subset of motifs**. Comparison between the contribution of all possible 5-mer motifs (*n* = 1024) on exon skipping (horizontal axis) and PAS readthrough (vertical axis). Upstream (left), internal or terminal exon (middle), and downstream (right) regions are depicted. Motifs showing significant Spearman correlation with altered exon skipping or readthrough are depicted in red. *DIDO* mutants show significant correlation for all regions. Inset numbers show Pearson correlation coefficient between PAS and exons. Note the reduced correlation in E3+4 for the downstream region. Random controls were derived from a 5 × 1000 random sample. Individual 5-mer motifs are annotated in supplementary figures S6-S8

To directly test the relation between the behavior of internal exons and PAS, the Spearman correlation coefficients for the two types of exons were compared (Figure 6, supplementary figures S6C–S8C). No significant correlation was found in random controls. All regions in E16 and E3+4 however showed overall positive correlation, indicating comparable behavior of internal and terminal exons. In general, large groups of related 5-mers, instead of a few specific motifs, controlled both exon skipping and PAS readthrough. The sequence motifs thus identified were very similar to those found to control splicing [19], with a strong effect of GC-rich and T-rich 5-mers. Correlation of downstream regions in E3+4 was weak – but still significant – as compared to upstream regions and exons, as well as compared to the E16 mutant (right panels). This comparatively weak correlation can be attributed mainly to E3+4 PAS behavior (vertical axis, supplementary figure S7C), in agreement with the low number of significantly contributing 5-mers identified in this region (supplementary figure S7B). In conclusion, internal and terminal exons were controlled by a similarly distributed motifs in E16, while the pausing-responsive E3+4 mutant may use common UTR-based 3ʹ end signals to process PAS. This divergent behavior again mirrors the way in which the *DIDO* mutants affect 3ʹ SS selection [19].

### Skipping and readthrough occur together

Previous evaluation of exon skipping showed that tandem 3ʹ UTRs, for example in the GAS2L1 gene, are susceptible to alternative processing in *DIDO* mutants [19]. To evaluate the behavior of 3ʹ gene ends without tandem organization, we analyzed intergenic regions downstream from unique UTRs. Also in genes bearing a single terminal exon, RNA sequencing showed slippage to downstream PAS in *DIDO* mutants (supplementary figure S9-S12). The scope of readthrough and location of downstream PAS again were flanked by TRSM, in agreement with previous results.

**Figure 7.**
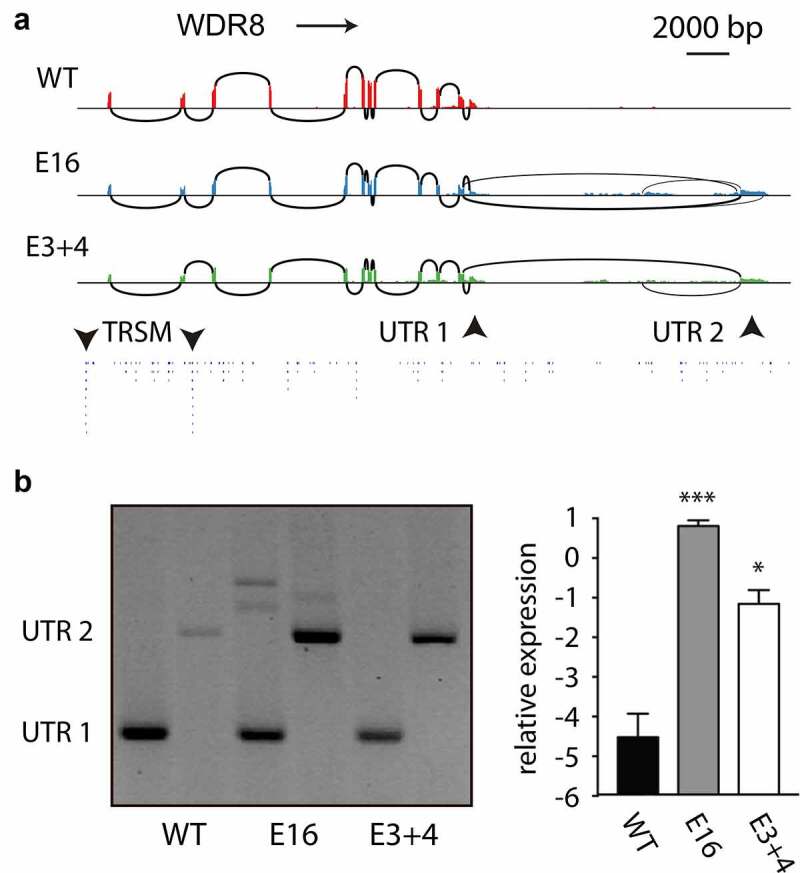
**Concurrency of exon skipping and readthrough**. As an example of altered processing of terminal exons, the *WDR8* gene was analyzed. (a) RNA sequencing indicated skipping of the terminal exon of WDR in E16 and E3+4 but not in WT controls. A cryptic alternative terminal exon outside the *WDR8* gene is used in the *DIDO* mutants. (b) Normal and (c) quantitative RT-PCR confirmed the sequencing data (***: *p* < 0.001; *: *p* < 0.05). Thus, while reduced splicing of internal exons leads to exon skipping in E16 and E3+4, the same genetic defect causes readthrough of terminal exons when no downstream alternative exon is available. In extreme cases, transcription may encounter a downstream 3ʹ SS in an adjacent gene before the upstream 3ʹ SS and PAS are processed, giving rise to gene fusions (supplementary figure S13)

Since a proportion of regions downstream of genes contained no TRSM (Figure 1), transcription could proceed over a long distance and into adjacent genes. The polyAsite database indeed indicated some PAS far downstream from described genes (supplementary figures S2-S4), and RNA sequencing revealed transcripts that continued for a comparable distance (Figure 7). In the *WDR8* gene, sequencing showed skipping of the terminal exon in E16 and E3+4 but not in WT controls. Normal and quantitative RT-PCR confirmed the sequencing data. Since the region downstream of *WDR8* bears only poorly defined TRSM, inefficient 3ʹ SS processing could rapidly result in transcriptional readthrough, up to a region where an intergenic 3ʹ SS is recognized. In the readthrough transcript, the original 3ʹ end is excluded by exon skipping while the alternative 3ʹ SS and PAS are processed together (Figure 7(b)). Thus, if transcription proceeds to far beyond the 3ʹ SS, splicing of the terminal intron no longer is efficient and the associated PAS is processed poorly.

If a downstream tandem gene is sufficiently close, skipping of the upstream UTR may give rise to fusion to an internal exon. RT-PCR of two examples identified by massive sequencing confirmed this possibility (supplementary figure S13). In such extreme cases, PAS associated with the upstream gene are bypassed. When a newly transcribed downstream 3´ SS becomes available, it will be favored due to proximity to Pol II.

## Discussion

Although the use of non-canonical PAS is commonly observed in vertebrates, our understanding of APA is limited. Most effort has gone into the study of cis-acting elements immediately surrounding PAS [3], even though distant signals are known to contribute to polyadenylation [36,37]. For example, polyadenylation is coupled to recognition of the preceding 3ʹ SS for a subset of transfected model genes [38]. A link between splicing and polyadenylation has been suggested for tandem terminal exons and intronic PAS, but was ignored for common forms of APA [10]. We thus explored the hypothesis that a wider gene architecture, in particular the upstream 3ʹ SS, has a cell-wide impact on APA. Genetic manipulation of splicing has proven difficult and in general is incompatible with cell survival; gross deletion of splicing factors leads to a number of defects, including suppressed transcription of the 3ʹ end of genes [39]. We avoided these so-called transcriptopathies by using two different mutants of the *DIDO* gene, which cause SFPQ redistribution but not a complete loss of function. For splicing, this means that reduced overall recruitment produces a surplus SFPQ pool available for processing of kinetically advantageous 3ʹ SS [19]. In agreement with a spatial SF distribution model [40], exons upstream from TRSM benefit most from this pool of free SFPQ [19]. Here, we analyzed the massive sequencing data from *DIDO* mutants while focusing on APA.

Analysis of NET-seq data showed TRSM enrichment on the 3´ flank of RNA Pol II spikes and peaks, confirming the contribution of TRSM to transcriptional pausing. A proportion of NET-seq signals were found some distance downstream from known genes, in the so-called “junk DNA”. Transcription thus seems to proceed well beyond known gene boundaries. Accordingly, TRSM distribution at 3ʹ ends (Figure 1(a)) already suggests that no sharp gene boundary is genetically defined. The adaptor protein DIDO3 followed a distribution comparable to Pol II at 3ʹ gene ends; we thus compared *DIDO* mutants to wild-type controls, determining readthrough levels based on RNA-seq reads upstream and downstream of PAS [41].

We have previously shown that excess exon skipping in *DIDO* mutants is caused by decreased recognition of 3ʹ SS [19]. While 3´ SS ablation of a model gene abrogates the downstream PAS *in vitro* [12], the *DIDO* mutants that reduced 3´ SS efficiency *in vivo* had a comparable impact on polyadenylation. For splicing, our data indicated no arbitration between 3ʹ SS, but exposed a limited window of opportunity which closes once transcription proceeds. Although multiple PAS typically share a single upstream 3ʹ SS, APA showed the same behavior. For APA, downstream slippage was observed, and typically continued until transcription reached a potential pause site (supplementary figures S8-S10). As previously observed for 3ʹ SS [19], PAS analyses produced a group for which readthrough increased and another for which it was reduced in *DIDO* mutants. The group of PAS showing reduced readthrough as compared to controls underscored the influence of TRSM. Thus, exon inclusion and PAS processing were controlled by a common group of TRSM, identified as Pol II pausing signals by NET-seq. In contrast, PAS lacking TRSM showed slippage toward downstream alternative sites. TRSM nonetheless do not seem to provoke a full stop, as indicated by tandem NET-seq peaks; pausing likely collaborates with known mechanisms such as the “torpedo” model [2].

Reduced 3´ SS processing had a combined effect on shortly spaced tandem genes. Here, transcription proceeded into adjacent genes and produced splicing-dependent gene fusions. Thus, the intermittent PAS is transcribed but fails to be recognized by the PA apparatus. Gene fusion becomes irreversible as soon as a downstream 3´ SS is used; the window of opportunity for the upstream sites closes definitively and the intergenic region is spliced out as an intron.

**Figure 8.**
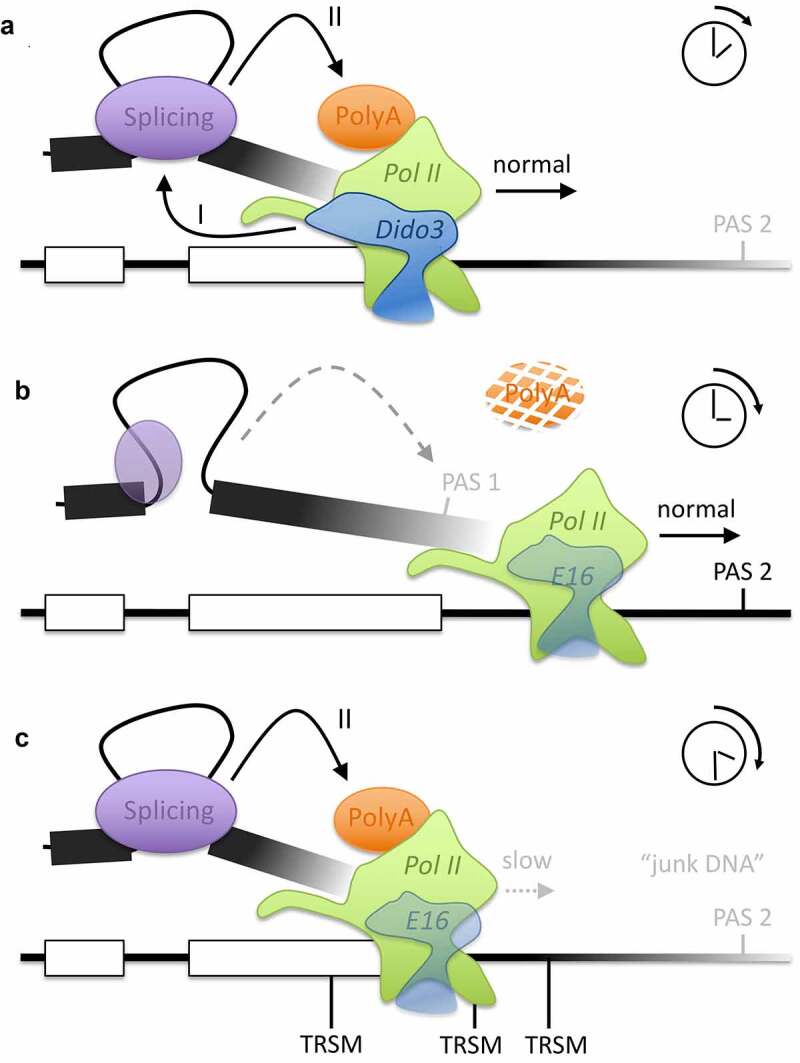
**Model for interplay of transcription, splicing, and polyadenylation**. (a) Under normal conditions, sufficient DIDO3 promotes recognition of the upstream 3´ SS (i) and thereby PAS processing (II). (b) Diminished or defective DIDO3 reduces 3´ SS processing with normal transcription speed, increasing slippage to downstream PAS and readthrough. (c) TRSM promote Pol II pausing, which increases the time for 3´ SS processing and thereby overcomes slow SF recruitment. *DIDO* mutation favors usage of PAS located close to TRSM, which may locate to a wide region between the 3´ SS and “junk DNA” downstream of genes

One question that remains is how a single upstream 3ʹ SS can select among multiple downstream PAS. The differential behavior of the two *DIDO* mutants may relate to such a selection mechanism. While the two *DIDO* mutants produced the same type of defects with respect to PAS readthrough, their sensitivity and response to transcriptional pausing diverged noticeably. In particular, TRSM located inside the terminal exon affected APA in E16 and E3+4 in a different manner. Importantly, these TRSM are located downstream of the 3´SS but upstream of the PAS, and thus are transcribed by Pol II before core PA signals become available. Only the latter mutant, partially functional and responsive to TRSM [19], was able to use UTR-based pause signals to promote PAS processing (Figure 5). The *DIDO* mutant lacking SFPQ binding was unable to benefit from pausing upstream of PAS. Pol II pausing may thus control APA by extending the time window during which the 3ʹ SS is visible before transcription of core polyadenylation signals (Figure 8). In conditions where APA rapidly changes, for example stem cell differentiation, DIDO3 expression levels indeed are very dynamic [22]. APA may thus involve a single 3ʹ SS but the variable capacity to recognize this 3ʹ SS and local modulation of transcription speed lead to diverse PAS selection. In its essence, 3ʹ SS recognition seems to define a variable time window during which RNA Pol II proceeds with transcription before activating the ability to decipher polyadenylation signals.

In our model, TRSM may also promote usage of weak non-canonical PAS, if these are close to – paused – RNA Pol II at the moment when the 3ʹ SS reaches readiness. Accordingly, our data showed increased usage of AAGAAA-based signals, which have no activity *in vitro* but are frequently found upstream of canonical PAS *in vivo* [3,29]. These AAGAAA-based PAS did not increase Pol II pausing by themselves (Figure 2) but appeared to be activated by distant TRSM in our mutants. The increased use of AAGAAA-based signals in *DIDO* mutants suggests that the role of the PAS core is more passive than commonly believed, explaining why even very degenerate signals – but flanked by downstream TRSM – support polyadenylation *in vitro* [42].

## Materials and methods

### Identification of short biased sequences

A biased sequence was defined as a 12 base-pair genomic window containing at least 9 thymidine residues. To count biased sequences, a sliding window of 12 bp was moved across all genes in the mm10 annotation, previously divided into introns, internal exons, terminal exons, and 5 kilobase regions downstream of genes. Overlapping features were filtered out. Whenever a hit on a biased sequence was recorded, the sliding window was moved to the end of the repeat, in order to prevent repeated counting of the same repeat. Finally, frequencies were calculated dividing repeat count by feature length in kilobases.

### RNA sequence analysis

RNA Pol II pause sites were evaluated on the basis of available NET-seq data (PRJNA356303) [31]. CHIP-seq data for DIDO3 (PRJNA33599) were published before [24]. Reads were aligned to the mm10 genome assembly with BWA MEM version 0.7.17 [43] and peak summits were called with MACS2 version 2.2.7.1 [44]. As MACS2 does not assign a strand, summits were expanded to 80 basepair regions (twice the average peak width) and strandedness was assigned by counting overlapping reads on each chromosome strand. Regions intersecting with internal (not terminal) exons or containing sequencing adaptors were removed, and remaining regions were used for analysis of sequence composition (full region) and k-mer quantification (3´ half of the region). Raw reads were visualized in the Integrative Genome Viewer [45], and T-rich genomic regions were identified by searching for the motif “TTTT”. Since the Integrative Genome Viewer considers overlapping motif as individual hits, a repetition of 5 consecutive Thymidines is represented as two hits in the figures.

For quantitation of exon skipping and PAS readthrough, the public PRJNA476070 dataset was used [19]. Paired RNA-seq reads were aligned against the mm10 genome assembly with Tophat2 version 2.1.0 [46]. Exon skipping was defined as the number of Tophat2 junctions spanning each exon, divided by the expression in FPKM. To determine readthrough, a window of 275 basepair upstream and downstream of each PAS in the PolyASite database [8] was used to quantify the number of mapped reads. For PAS located within 275 basepairs of each other, only the most highly used PAS was considered. Readthrough was defined as the number of downstream reads divided by the number of upstream reads. Altered exon skipping or readthrough events were identified as those that were significantly different (p < 0.05) in a two-tailed t-test, comparing triplicate mutant samples to the same number of WT controls. As the t-test is intrinsically conservative and our data showed a large degree of interdependence, no p-value correction was carried out.

To establish correlation between local motif frequency and readthrough, a window spanning the region between 200 bases upstream from the 3ʹ SS and 200 bases downstream from the PAS was established. For skipped exons, a genomic window was established using the exon boundaries as reference points. For each window, the frequency of each 5-mer motif was established. Overlapping instances of the same motif were corrected for, so that a repetition of six consecutive bases counted as 1.2 events. For each 5-mer, this yielded a list of local frequencies paired to fold change in skipping (for exons) or readthrough (for PAS), from which a Spearman correlation was calculated. The number of significantly altered exons or PAS equals the size of one list, and is reported in the figures. Finally, Spearman correlation of each 5-mer (1024 data points) was plotted against the normalized frequency across all samples.

### RT-PCR confirmation

Mouse embryonic fibroblasts (MEF) were cultured in DMEM supplemented with 10% fetal calf serum and antibiotics. MEF lacking *DIDO* exon 16 (E16) or *DIDO* exons 3 and 4 (E3+4) have been described [19,23]. For RNA isolation, MEF were grown to 80% confluence, washed once in PBS, and lifted by trypsinization. After washing again with PBS, total RNA was isolated from approximately 5 · 10^6^ cells with a SPLIT RNA extraction kit (Lexogen, Austria) using the manufacturer´s protocol. RT-PCR amplifications on 1 μg total RNA were performed using a Verso One-step RT-PCR Kit (ThermoFisher, Waltham, MA). Primer sequences are provided in the supplementary documentation.

## Supplementary Material

Supplemental Material
