# Supplementary material for: Interplay between splicing and transcriptional pausing exerts genome-wide control over alternative polyadenylation
Source: Transcription. 2021 Aug 7;12(2-3):55–71. doi: 10.1080/21541264.2021.1959244 (PMC8555548; doi:10.1080/21541264.2021.1959244)
Supplement: Supplemental Material [file KTRN_A_1959244_SM8057.zip › downloadFromZipFile.pdf]

Supplementary information for:

Interplay between splicing and transcriptional pausing exerts genome-wide control over alternative polyadenylation

Carmen Mora Gallardo, Ainhoa Sánchez de Diego, Carlos Martínez-A, and Karel H.M. van Wely

RT-PCR Primers used in this study:

WDR8 FW:	GCTGTTTGTGGTACTAGAGC
WDR8 RV1:	AGACAGAAGTGGTCCTTACT
WDR8 RV2:	TGCCTTAAATGTGCTGTCAA
CUEDC1 FW:	GATCTGGGAAGTCTGGACAG
CUEDC1 RV:	ACAAATGGGGTTTGTGGCTG
MRPS23 FW:	CGTGTTCTCCCGGACGAG
MRPS23 RV:	GCTCTCTCAGGGGTGGAAAG
RAD23A FW:	AATTCCAGGAAGCCCTGAGC
RAD23A RV:	CTTCAGCTATGGCACCAACC
DAND5 FW:	TTTAGTCCGTACGGCGCAGG
DAND5 RV:	GAACAGCGGCCAAAACAGAG

Supplementary figures

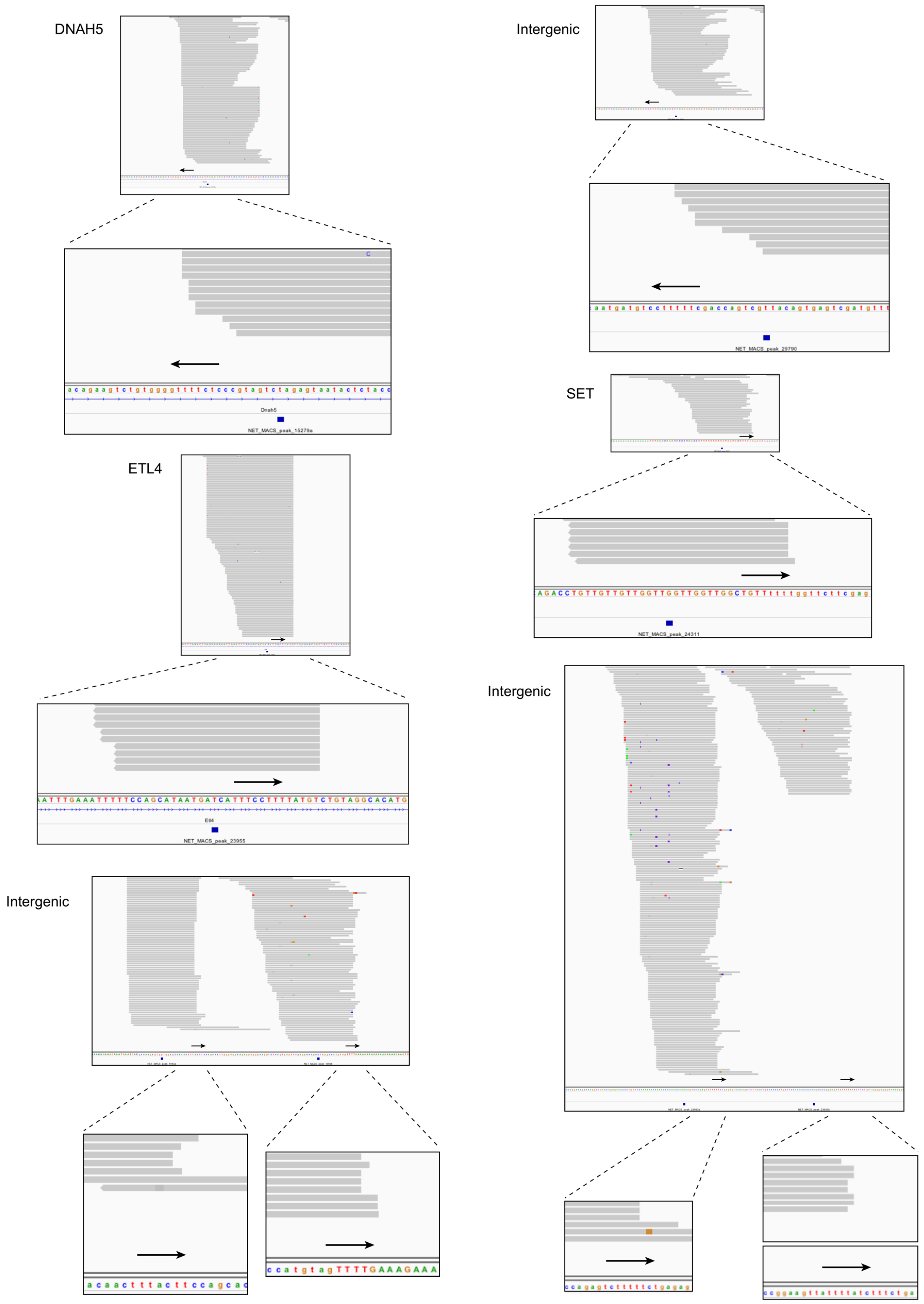

Supplementary figure S1. Downstream flanks of NET-seq peaks frequently bear TRSM. IGV screenshots are shown, arrows indicate TRSM, and chromosome strands were adjusted to peak orientation. Genes are indicated to the left. Single and tandem peaks were observed.

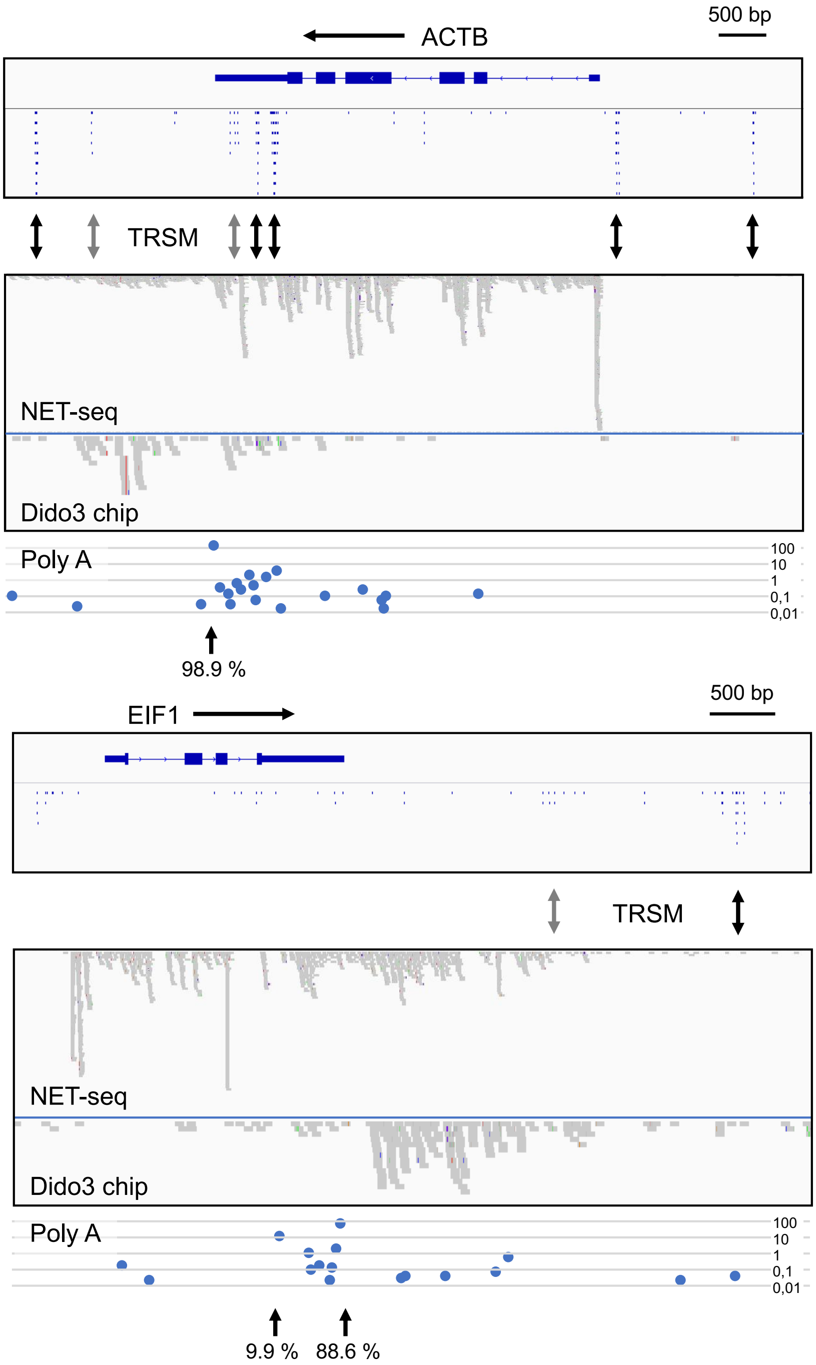

Supplementary figure S2. Localization of NET-seq and DIDO3 peaks relative to T-rich elements and PAS usage. TRSM (upper panel) are identified by searching for the "TTTT" motif in IGV. NET-seq and CHIP-seq data (middle panel) show raw alignments. PAS usage (lower panel) is derived from the PolyASite database and normalized to a total of 100% over the shown genomic region and gene strand. Note the accumulation of Pol II and DIDO3 upstream of strong TRSM (black arrows), and the relative location of canonical PAS.

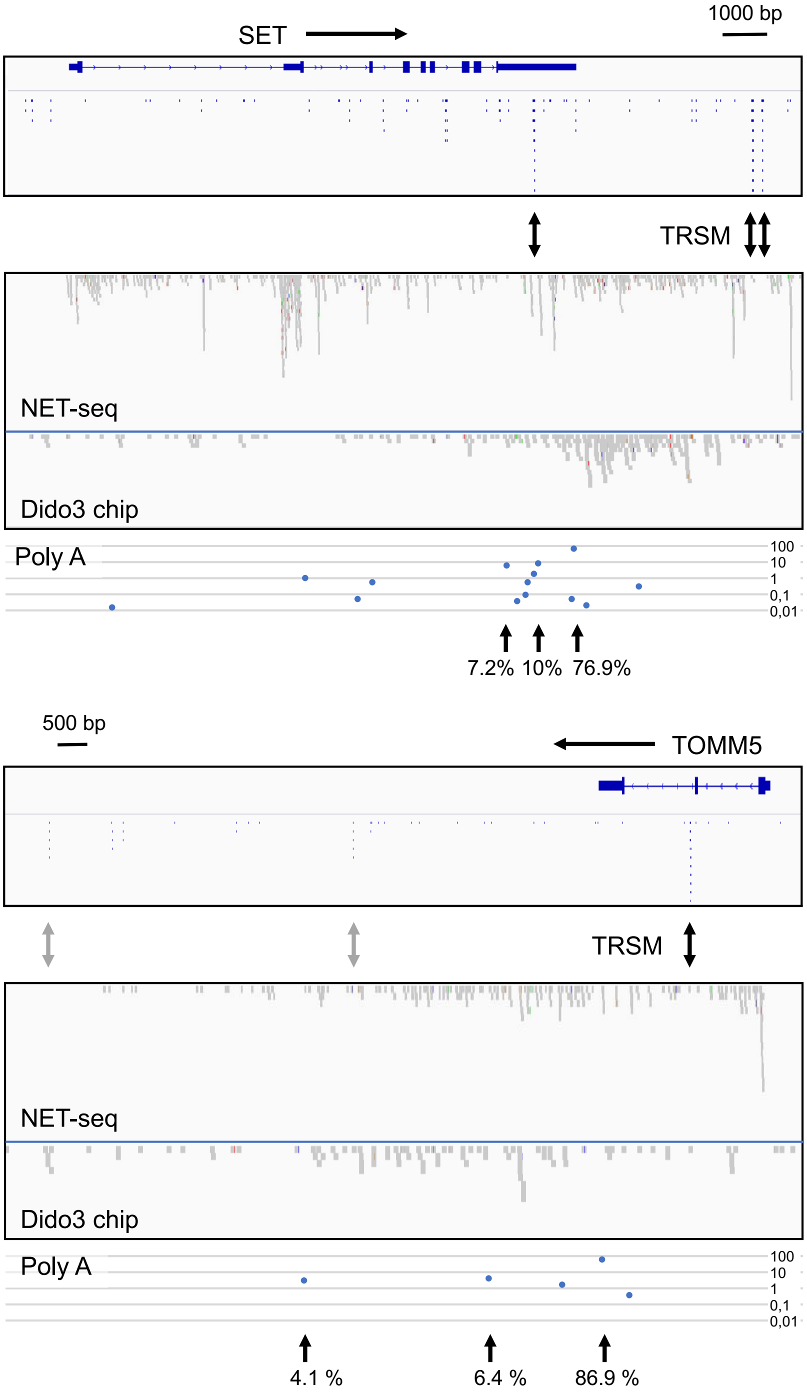

Supplementary figure S3. Localization of NET-seq and DIDO3 peaks relative to TRSM and PAS usage. Panels are marked as in supplementary figure S2. Note the formation of spikes in NET-seq in the SET gene, as compared to broad peaks in highly transcribed genes *ACTB* and *EIF1* (supplementary figure S2). Shorter TRSM (*TOMM5*) allow for partial readthrough, leading to Pol II signals downstream of canonical PAS.

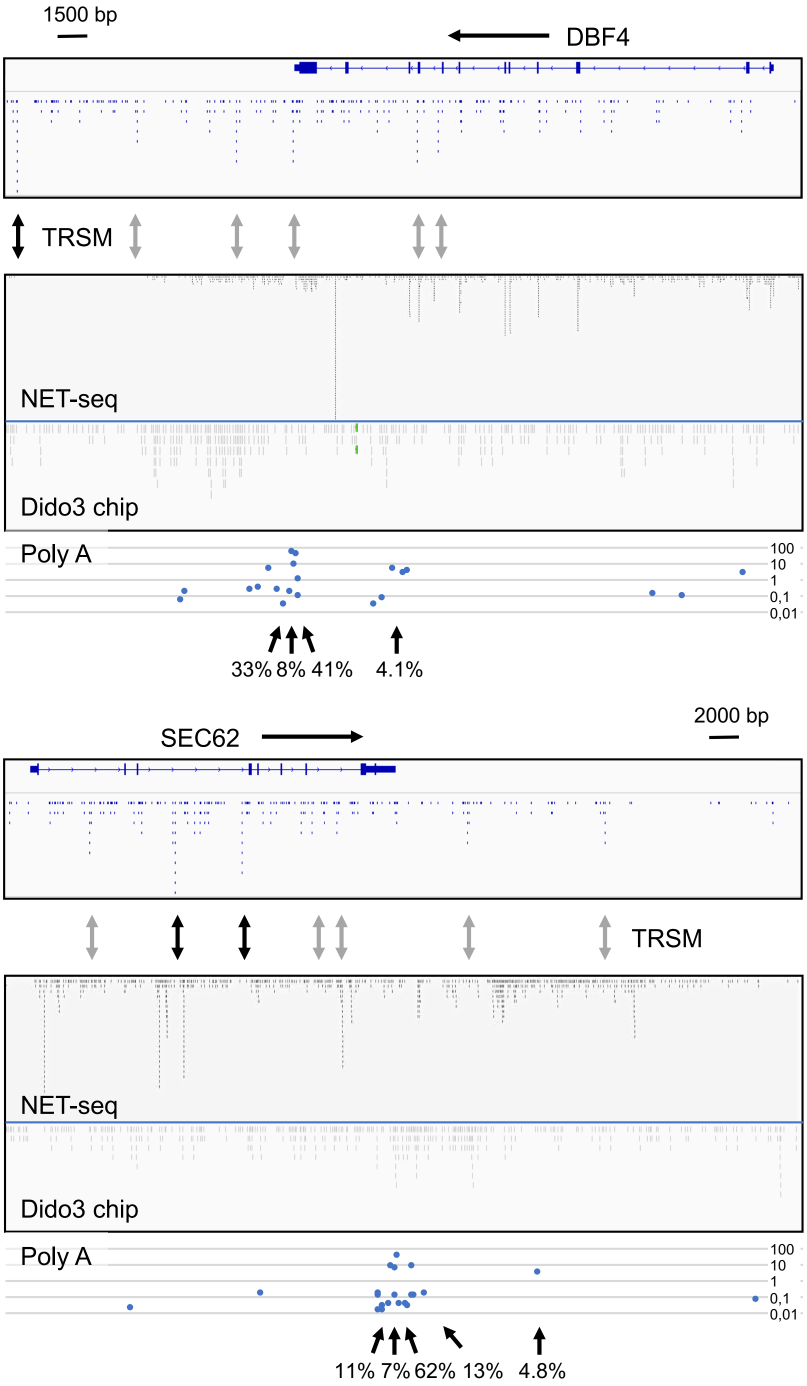

Supplementary figure S4. Comparison of NET-seq and DIDO3 accumulation in the absence of strong TRSM. Panels are marked as in supplementary figure S2. Although intragenic pausing was found, weaker TRSM surrounding the 3´ gene end were associated with partial readthrough and activation of downstream PAS.

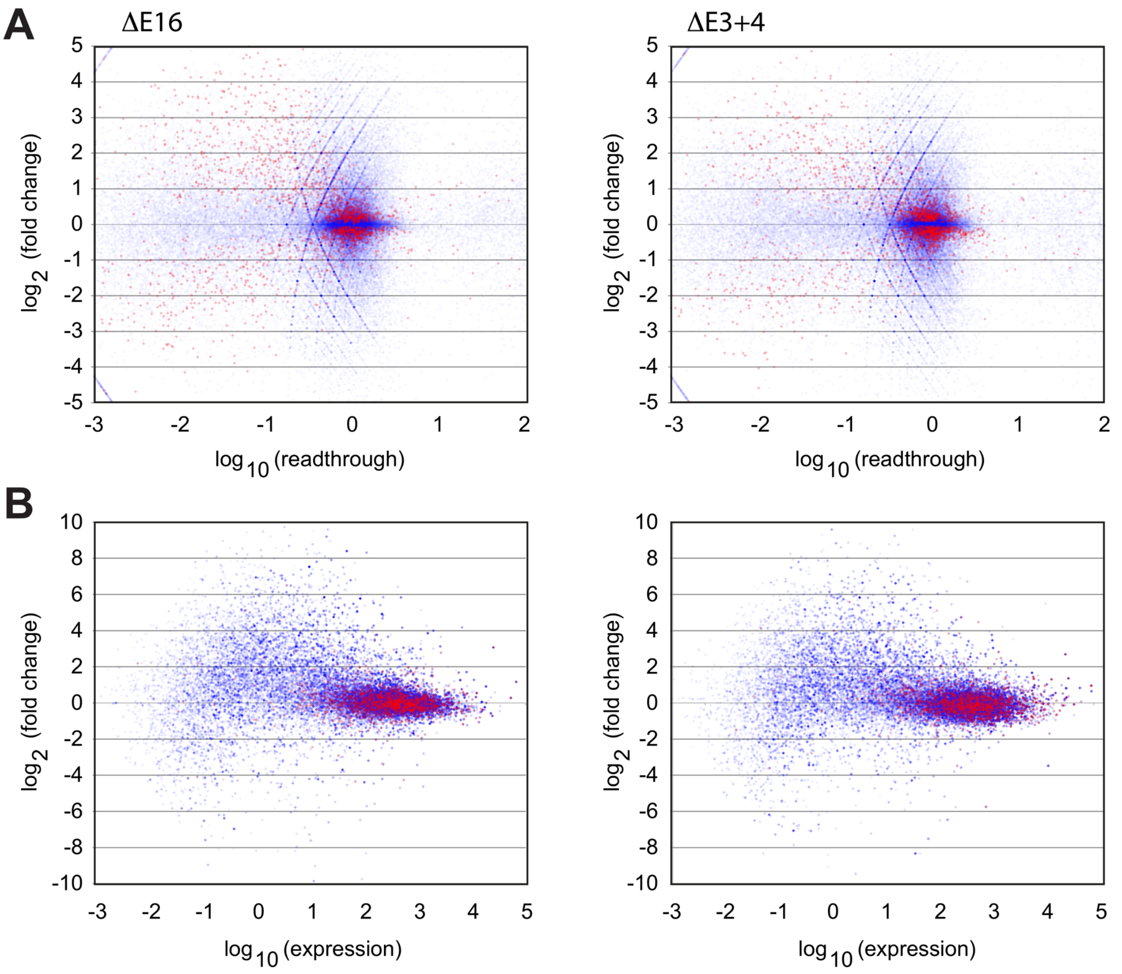

Supplementary figure S5. General classification of PAS. (A) Average readthrough of all PAS (blue) in wild-type samples was determined (horizontal axis), and plotted against changes in readthrough (vertical axis) in *Dido* mutant MEF. PAS showing significantly altered readthrough ($p<0.05$) are highlighted (red). Distribution of the two populations along the horizontal axis is comparable. (B) PAS showing significantly altered readthrough were mapped to the corresponding upstream exons, and their expression was plotted. Terminal exons undergoing altered readthrough in *DIDO* mutants (red) follow a distribution comparable to the general population (blue), and show no overall expression differences.

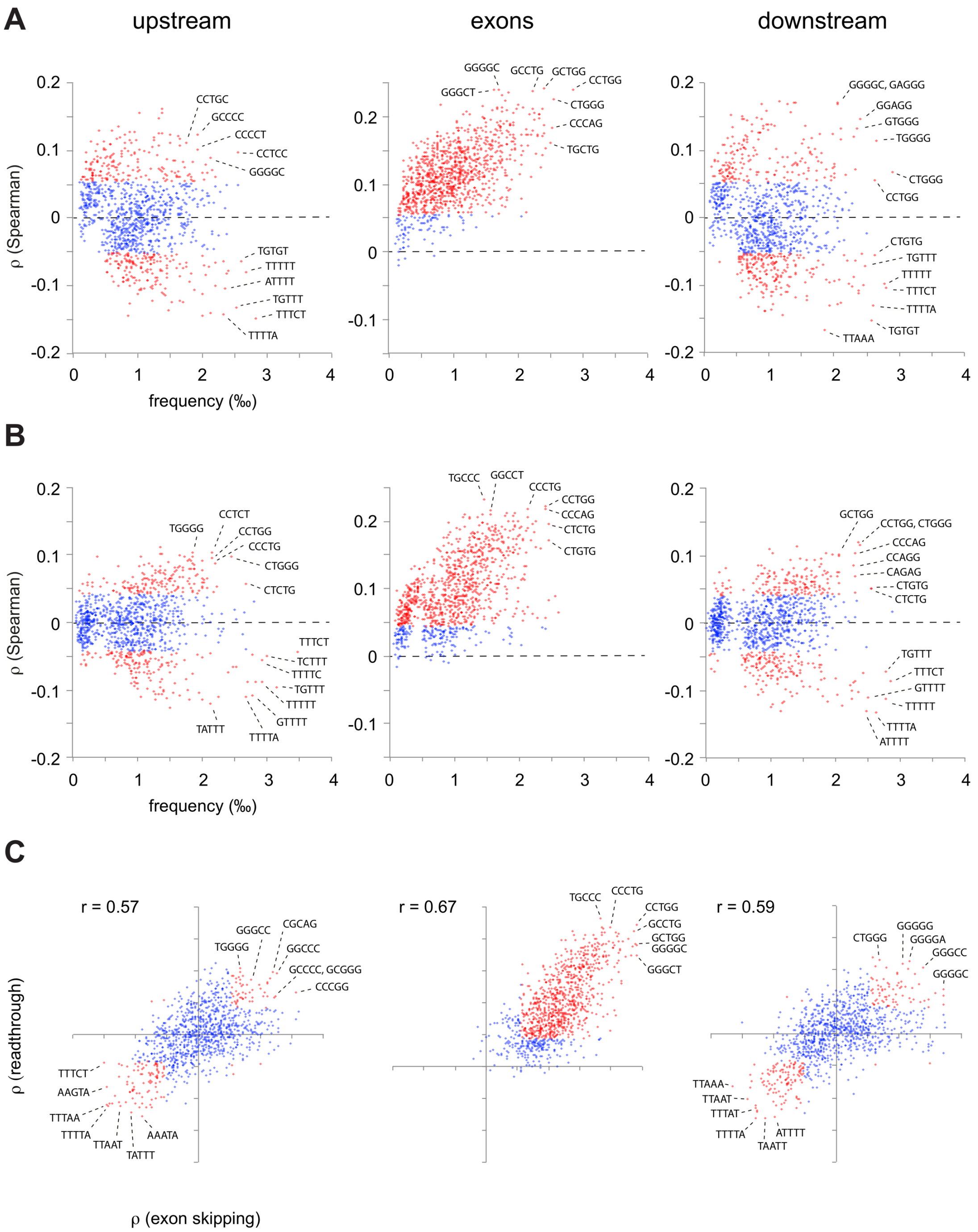

Supplementary figure S6. Sequence motifs involved in exon skipping and PAS readthrough in E16. The three genomic regions potentially contributing to significantly altered exon skipping (A) or PAS readthrough (B) were analyzed. The frequency of occurrence of 5-mer motifs (horizontal axis) is plotted against the correlation with readthrough (vertical axis). Motifs with a significant Spearman correlation are indicated in red. A subset of motifs showing significant correlation is indicated. The control population was derived from a random sample of 5x1000 PAS. (C) Comparison between motifs involved in exon skipping and motifs involved in readthrough. While 5-mers in all regions show significant Pearson correlation, no contribution of T-rich motifs was found for exonic regions.

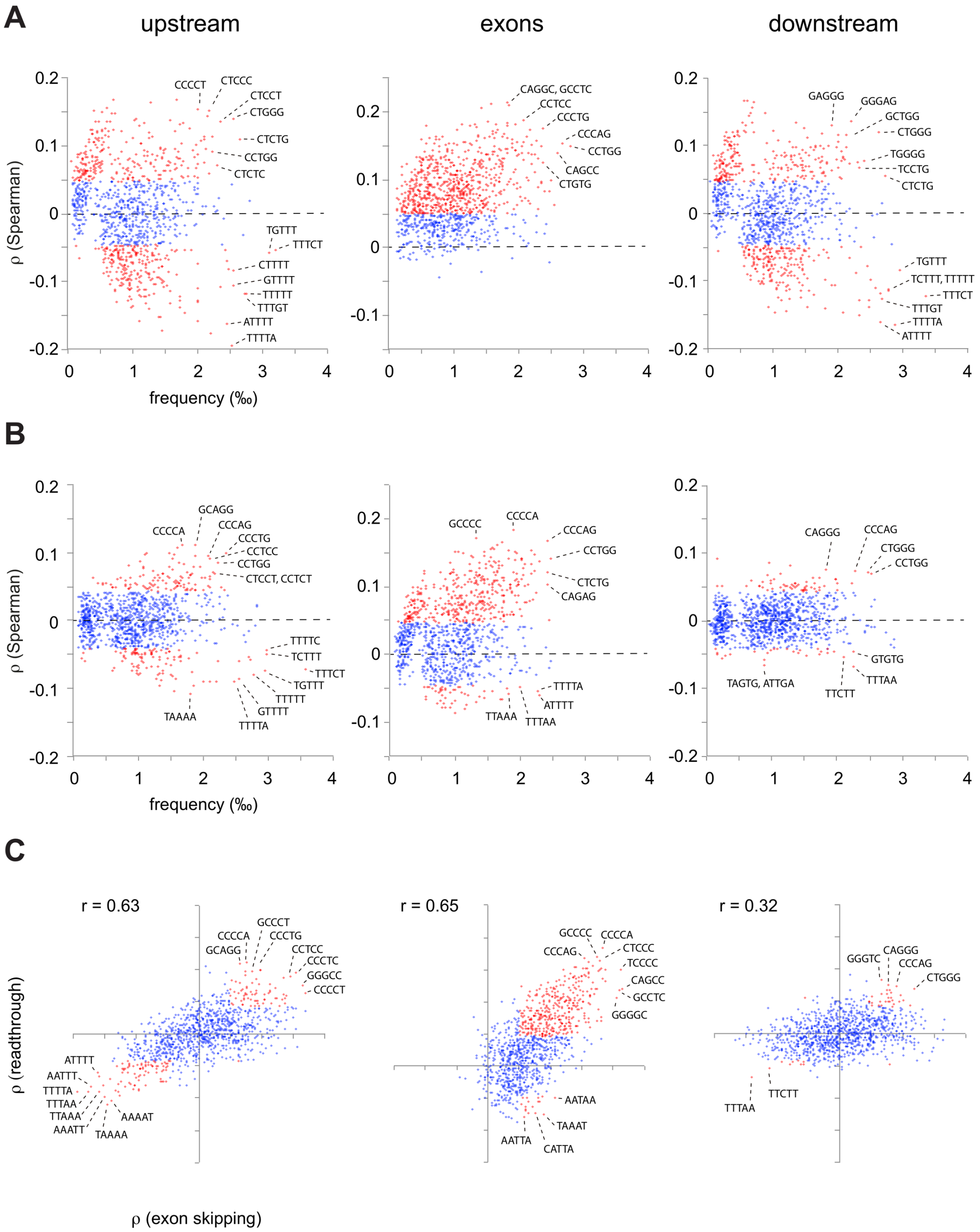

Supplementary figure S7. Sequence motifs involved in exon skipping and PAS readthrough in E3+4. Genomic regions were analyzed as in supplementary figure S6, using data for the E3+4 mutant. (B) In this mutant, a contribution of UTR-based T-rich motifs to PAS usage was observed. (C) Note the limited impact of 5-mers in the region downstream of PAS in this mutant, resulting in a comparatively weak Pearson correlation.

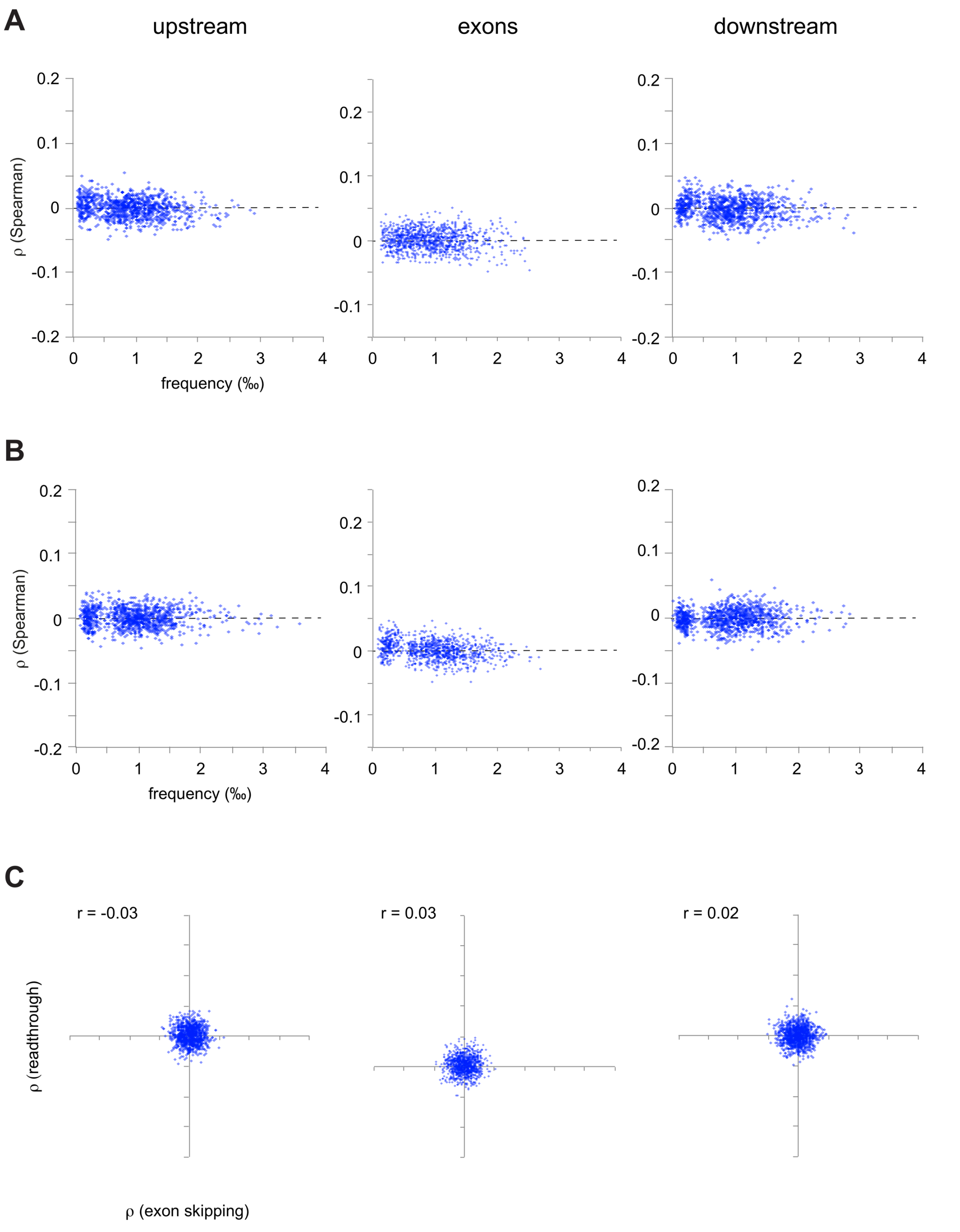

Supplementary figure S8. Random controls show no significant correlation between motifs involved in exon skipping and readthrough. Genomic regions were analyzed as in supplementary figure S6, using a random control population of 5x1000 exons. No motifs showing significant correlation were found.

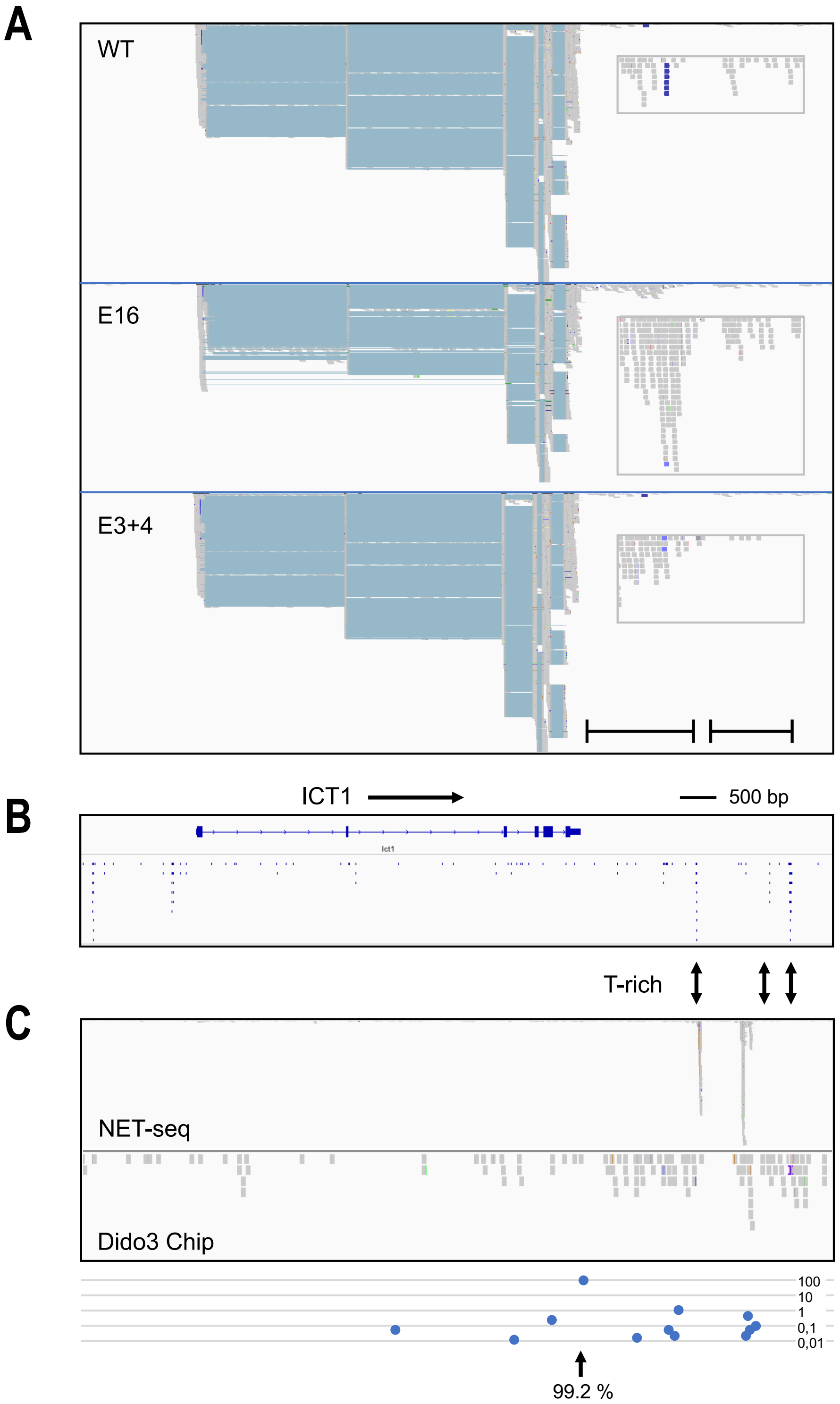

Supplementary Figure S9. Slipping towards downstream PAS in the *ICT1* gene. Comparison of (A) raw RNA sequencing reads, (B) genomic organization and TRSM, and (C) raw NET-seq reads, Dido3 CHIP-seq reads and WT usage of PAS. Total use of PAS in the region was normalized to 100%. Insets show amplification of RNA seq reads in the region affected by readthrough in the *DIDO* mutants. Note the relative location of NET-seq and RNA-seq reads.

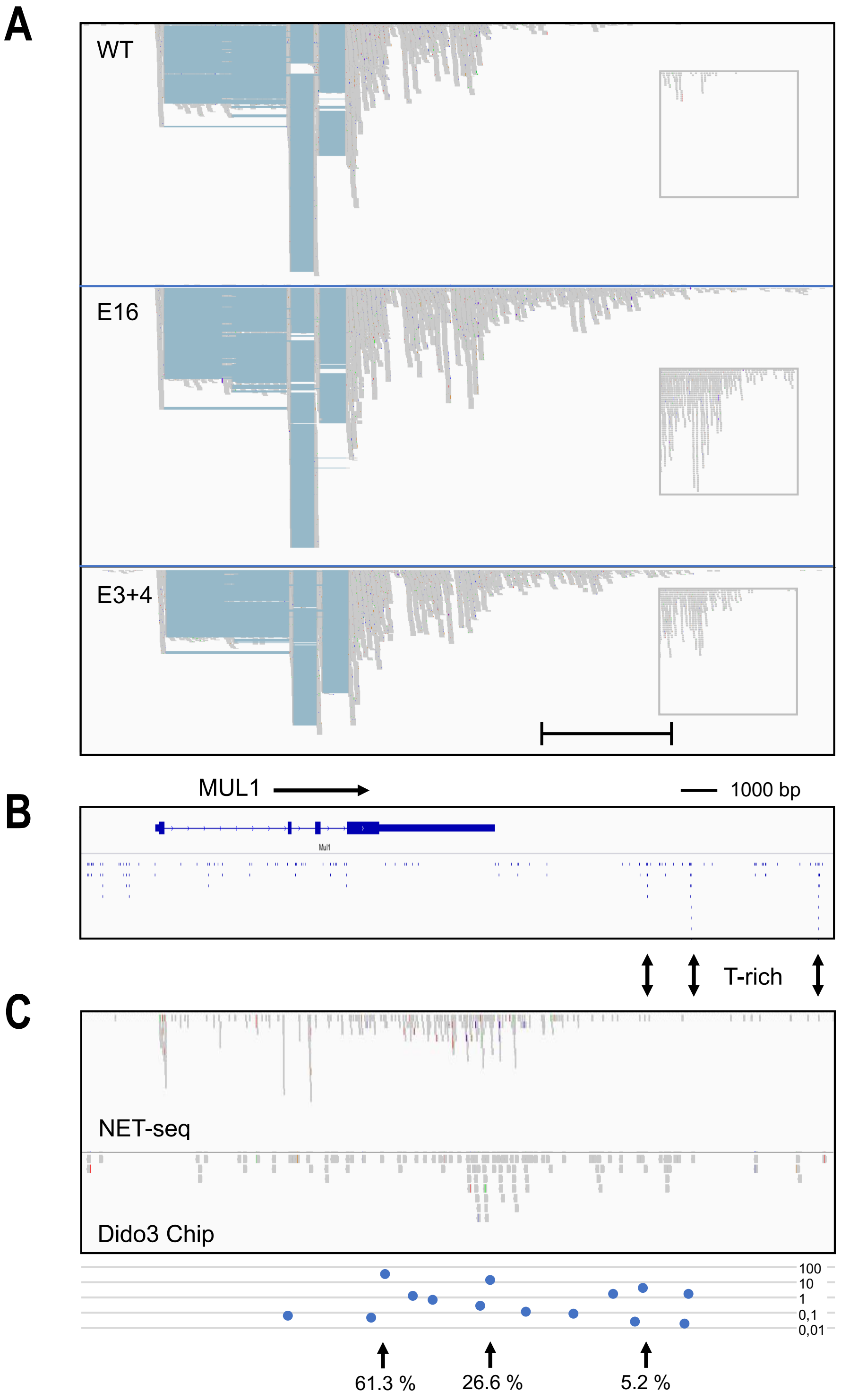

Supplementary Figure S10. Slipping towards downstream PAS in the *MUL1* gene. Features are depicted as in supplementary figure S9. NET-seq, CHIP-seq, and RNA-seq reads in the *DIDO* mutants extend up to the TRSM, several kb downstream from the gene.

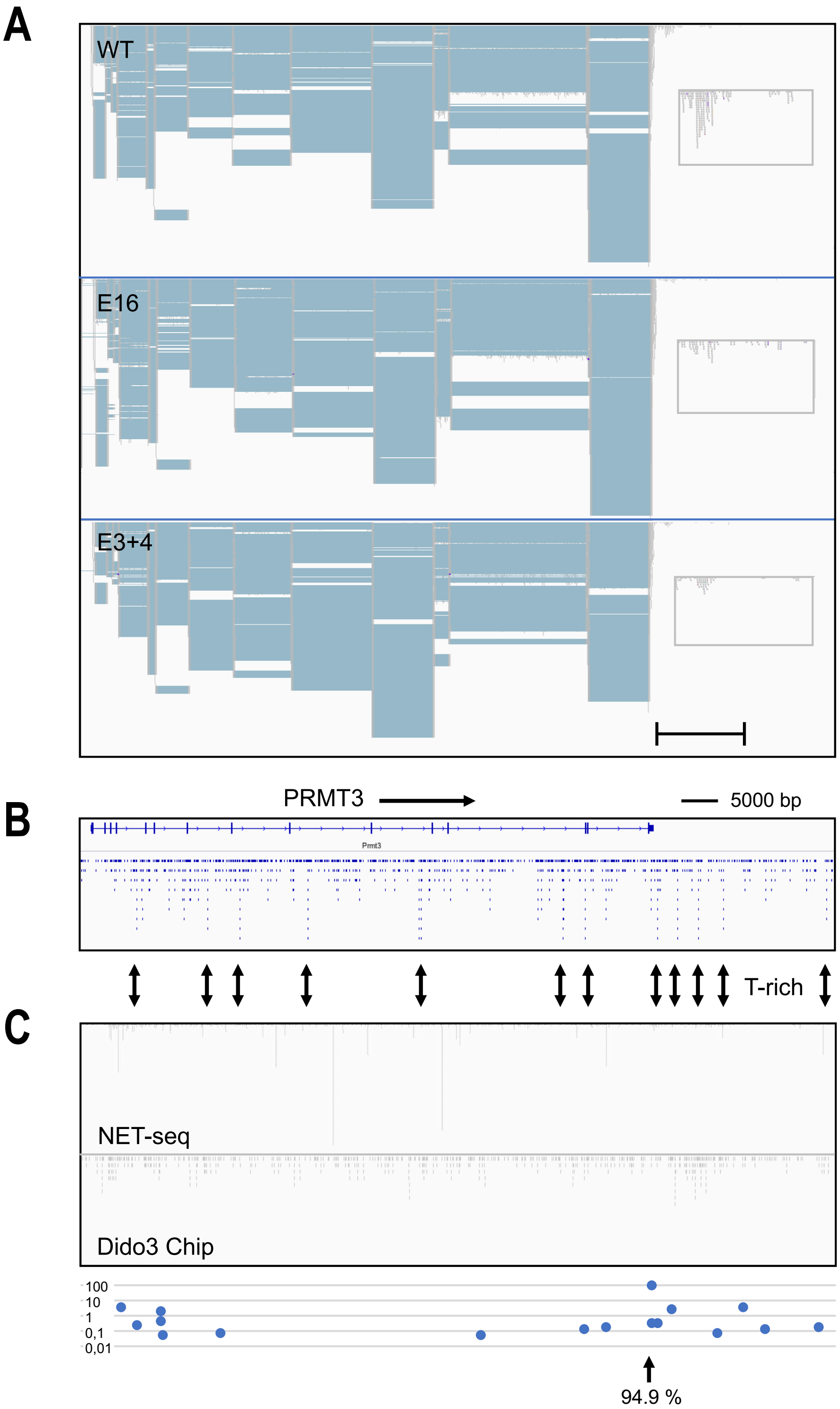

Supplementary Figure S11. Decreased readthrough in the *PRMT3* gene. Features are depicted as in supplementary figure S9. TRSM directly downstream from the canonical PAS suppress readthrough in the *DIDO* mutants; NET-seq shows a gene-wide (multiple pausing) distribution pattern, as compared to 3' end accumulation in *ACTB* and *EIF1*.

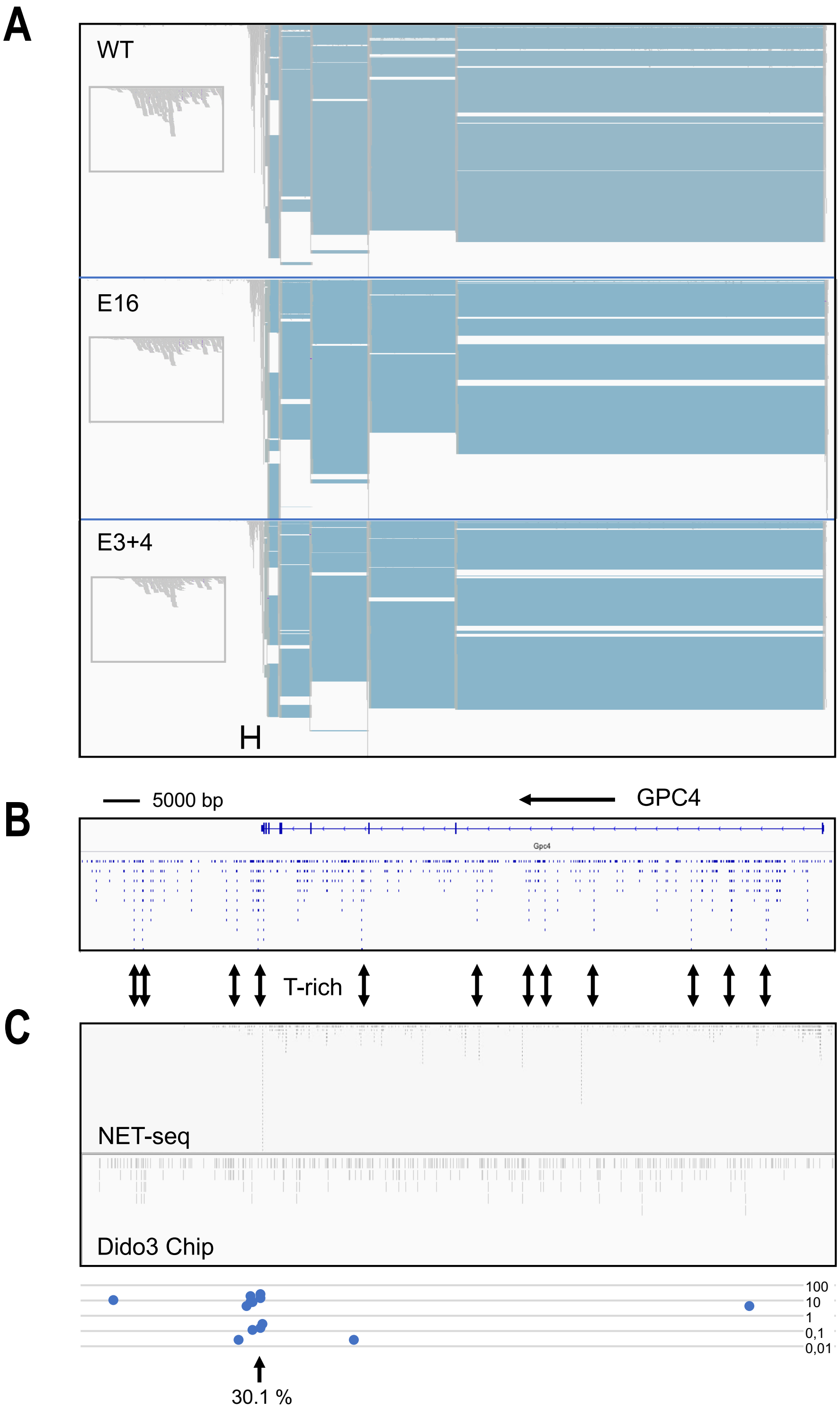

Supplementary Figure S12. Decreased readthrough in the *GPC4* gene. Features are depicted as in supplementary figure S9. TRSM directly downstream from the canonical PAS suppress readthrough in the *DIDO* mutants. Note the single dominant NET-seq peak on the PAS cluster.

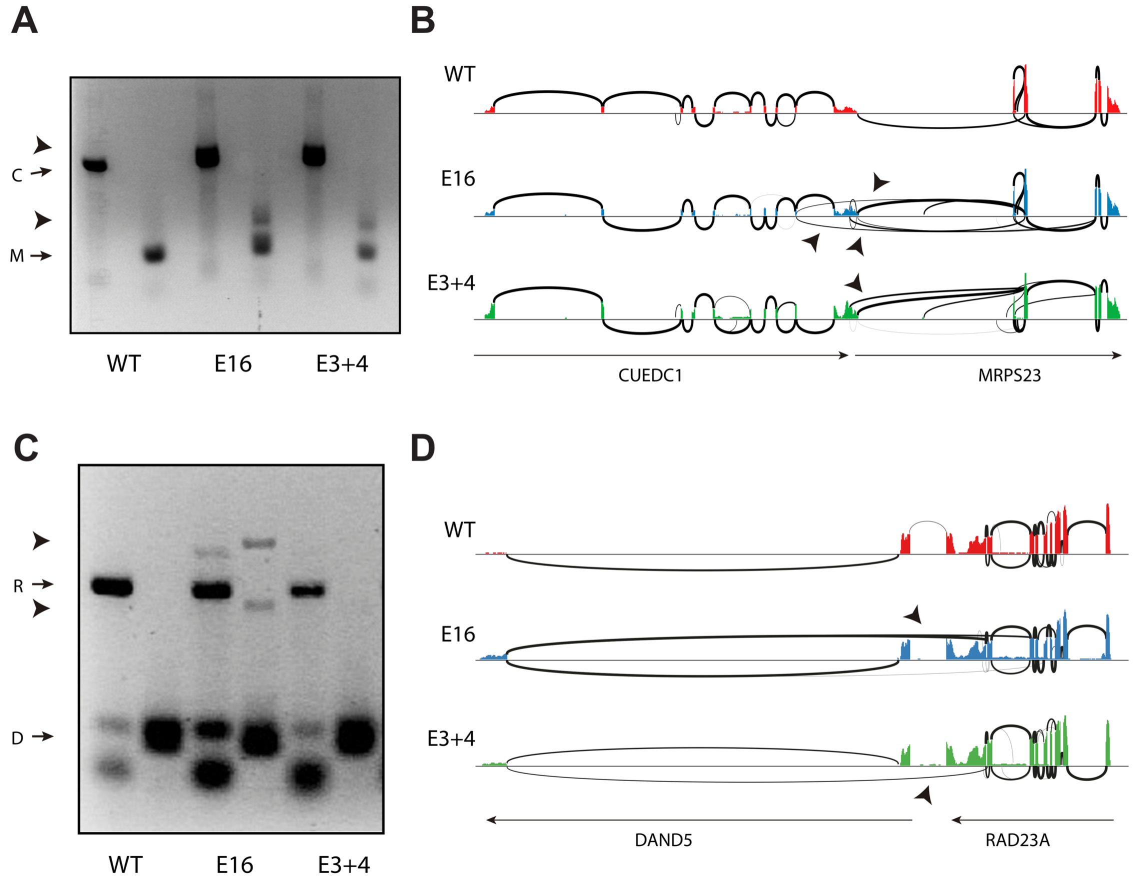

Supplementary figure S13. Further examples of gene fusion in *DIDO* mutants. RT-PCR (A) and Sashimi (B) analysis of the *CUEDC1-MRPS23* fusion. Arrows indicate internal, gene-derived PCR products and arrowheads indicate fusion products. Note that the larger fusion product is only slightly longer than the corresponding internal product. RT-PCR (C) and Sashimi (D) analysis of the *RAD23A-DAND5* fusion. Arrows and arrowheads (A,C) indicate internal gene-derived and fusion products, respectively. Note that the low level of *RAD23A* readthrough in E3+4 remains undetected under these conditions. RT-PCR products were cloned and verified by sequencing.
